# Antimicrobial Use and Veterinary Care among Agro-Pastoralists in Northern Tanzania

**DOI:** 10.1371/journal.pone.0170328

**Published:** 2017-01-26

**Authors:** Mark A. Caudell, Marsha B. Quinlan, Murugan Subbiah, Douglas R. Call, Casey J. Roulette, Jennifer W. Roulette, Adam Roth, Louise Matthews, Robert J. Quinlan

**Affiliations:** 1 Paul G. Allen School for Global Animal Health, Washington State University, Pullman, Washington, United States of America; 2 Department of Anthropology, Washington State University, Pullman, Washington, United States of America; 3 Department of Anthropology, San Diego State University, San Diego, California, United States of America; 4 Department of Sociology, Washington State University, Pullman, Washington, United States of America; 5 Institute of Biodiversity, Animal Health and Comparative Medicine, University of Glasgow, Glasgow, Scotland, United Kingdom; The University of Melbourne, AUSTRALIA

## Abstract

Frequent and unregulated use of antimicrobials (AM) in livestock requires public health attention as a likely selection pressure for resistant bacteria. Studies among small-holders, who own a large percentage of the world’s livestock, are vital for understanding how practices involving AM use might influence resistance. We present a cultural-ecological mixed-methods analysis to explore sectors of veterinary care, loosely regulated AM use, and human exposure to AMs through meat and milk consumption across three rural to peri-urban Tanzanian ethnic groups (N = 415 households). Reported use of self-administered AMs varied by ethnic group (Maasai: 74%, Arusha: 21%, Chagga: 1%) as did consultation with professional veterinarians (Maasai: 36%, Arusha: 45%, Chagga: 96%) and observation of withdrawal of meat and milk from consumption during and following AM treatment (Maasai: 7%, Arusha: 72%, Chagga: 96%). The antibiotic oxytetracycline was by far the most common AM in this sample. Within ethnic groups, herd composition differences, particularly size of small-stock and cattle herds, were most strongly associated with differences in lay AM use. Among the Arusha, proxies for urbanization, including owning transportation and reliance on “zero-grazing” herds had the strongest positive associations with veterinarian consultation, while distance to urban centers was negatively associated. For Maasai, consultation was negatively associated with use of traditional healers or veterinary drug-shops. Observation of withdrawal was most strongly associated with owning technology among Maasai while Arusha observance displayed seasonal differences. This “One-Health” analysis suggests that livelihood and cultural niche factors, through their association with practices in smallholder populations, provide insight into the selection pressures that may contribute to the evolution and dissemination of antimicrobial resistance.

## Introduction

Antimicrobial resistance (AMR) is a significant global public health concern [1–3] and the use of commercial antimicrobials (AM) in livestock is considered an important component of this problem [4–6]. AM use among small-holders in low-income nations is an understudied behavior [7–10] and is potentially a significant contributor to the emergence and transmission of AMR considering that small-holders produce 80% of the world’s food [11] with 40% of global agricultural value tied to livestock [12,13]. In Africa, AM use appears widespread but detailed studies of AM-related behavior are rare (but see [7, 14, 15]). Research over the last 40 years has suggested that AMR in East Africa is associated with human-animal contact, high levels of antibiotic use in small production systems, lack of withdrawal for human consumption of meat and milk products from recently treated animals, and frequent or less “prudent” AM use [9].

Antimicrobials are important tools for herd management, but the manner in which they are used varies with species, local conditions, and cultural practices. The large herds typical of pastoralist groups graze across considerable distances and may experience more exposure to pathogens resulting in greater motivation for AM use, thereby promoting the emergence and selection for AMR. When these herds are found in remote locations, professional veterinary care is probably limited [16]. In contrast, smaller “zero-grazing” herds that spend most or all of their life in a pen could experience less exposure to pathogens and resistant bacteria. Herd composition, such as the number of cattle and small-stock (sheep and goats) may also influence the frequency and type of AM administration. Consequently, herd characteristics, and cultural practices are important facets of our analysis.

Indigenous Tanzanians call antimicrobials “exotic medicines”, recognizing their external origins. AM use is now part of “traditional” practice, however, in that the oldest of our Maasai informants (≈60–75 years) could not recall a time before AMs. Globalization has made the medical technologies of large, specialized market economies (antibiotics) accessible to groups who are less inclined by tradition or exposure to employ medical specialists [17,18]. In northern Tanzania, this pattern is exemplified by the diffusion of AM products into lay use in remote and livestock-dependent communities.

Our study groups vary in their involvement with the three “sectors of healthcare” [19,20] that describe veterinary practice in northern Tanzania. The “professional sector” includes state certified veterinarians and livestock extension officers. Tanzanian veterinarians participate in an extensive network of district-level, government sponsored, livestock extension services and limited private practice. Livestock owners can contact local, government, or private “vets” for an affordable fee to treat sick animals. In some communities, members of this professional veterinary sector administer all AMs, and, given their training and incentives, should follow recognized standards for diagnosis, dosage, and course of treatment. Veterinarians also encourage observance of a “withdrawal” period after completing a course of AMs before consuming or selling animal products (about seven days for milk or 30 days for meat). The “popular sector” or lay sector of veterinary care includes household use of relatively unregulated, “over-the-counter” AMs with little or no professional involvement. Lay sector use of AM appears widespread among pastoralists in Africa [7,14,16] and in a large segment of our sample. Presumably, use of AMs in this manner is expected to result in higher prevalence of AMR compared to treatment by certified professionals following best practices [9]. Finally, the “folk sector” includes traditional healers (sometimes in addition to biomedicine). In northern Tanzania, livestock owners may seek out *laibon* (shamans) [21] or herbalists, but traditional healers do not appear to specialize in animal health nor are AMs used in the folk sector. Several Maasai herbal remedies, nevertheless, have antimicrobial properties that may be relevant to AMR [22], and traditional practice might be relevant for veterinary public health [23]. Hence, we examine self-reported use of traditional healers.

Cultural-ecological factors associated with livestock management and involvement in the professional and lay sectors of veterinary care are the focus of these analyses. Analyses contrast three ethnic groups—Maasai, Chagga, and Arusha—across a gradient of animal husbandry systems. Maasai tend large free-range cattle and small-stock herds kept for both consumption and sale. Chagga are primarily highland farmers with small zero-grazing herds that are maintained mainly for household dairy consumption. Arusha inhabit highland and lowland environments with husbandry systems that share similarities with Maasai and Chagga. We assessed a group’s integration of subsistence practices and professional and lay sectors with outcome variables estimating (1) loosely regulated lay use of injectable AMs, (2) consultation with certified veterinary professionals, and, (3) observance of withdrawal periods from meat and milk. Below, we present a series of qualitative vignettes and statistical models.

## Methods and Materials

As part of a larger study of AMR in northern Tanzania our team surveyed over 400 households across 13 localities (see Table 1) between 2013 and 2015. Design of the household survey was informed by a mixed-methods approach that combined qualitative and quantitative interviews. Beginning in 2012, we conducted formal qualitative key informant and focus group interviews [24] among Maasai, Chagga, and Arusha livestock owners and observed livestock management and veterinary care practices. We interviewed livestock extension officers and veterinarians in different communities to determine common practices and the course of professional veterinary training in Tanzania. We visited veterinary drug shops in Monduli, Simanjiro, Arusha City and Moshi districts, and interviewed attendants about AM sources, sales, and recommended usage. Unless otherwise indicated, our ethnographic descriptions (see Results and Discussion below) draw on these materials. See the ESM for a more detailed description of qualitative interviews.

**Table 1:** Attributes of study villages (N = 415 households).

Ethnicity	Site	*N*	Season	Year(s)	Elevation (m)
Maasai	Monduli	34	Wet&Dry	2013/15	1617
	Loibor Siret	45	Dry	2013/15	1334
	Terat	23	Wet	2013	1423
	Nadonjukin	45	Wet&Dry	2013/14/15	1395
	Loliondo	20	Dry	2013	1907
	Komolo	30	Dry	2015	1073
Arusha	Aremeru ward	51	Wet&Dry	2013/15	1553
	Loroi	33	Dry	2015	1183
	Meliot	34	Dry	2015	1253
Chagga	Masaera kati	27	Wet&Dry	2014	1012
	Masaera juu	23	Dry	2014	1234
	Mamsera chini	25	Dry	2014	1153
	Mamsera juu	25	Dry	2014	1646

In conjunction with qualitative data collection, we developed a survey instrument of over 200 items including a broad range of livelihood and health topics suitable for comparison with other behavioral science and public health projects in the region [25] (See ESM for household questionnaire). Many survey items were used previously in Kenya and Ethiopia [26,27] then modified for present purposes. Research assistants fluent in English, Swahili and Maa or Chagga were trained and employed for data collection. Key informants and survey participants were compensated 10,000 Tanzanian Shillings (TZS) (about $6.50 USD) for participation (up to 120 min). The study was reviewed and approved by Washington State University’s and Tanzania National Institute for Medical Research’s Institutional Review Boards. Participant consent was verbally attained given rates of illiteracy within the study populations. Verbal consent was documented in a separate data file indicating informant ID numbers, agreement to participate, and payment/receipt of informant fees. This consent procedure was approved by Washington State University’s Institutional Review Board. Research permits were issued by the Tanzania Commission for Science and Technology, Tanzania Wildlife Research Institute, and by regional, district, and ward offices within Arusha, Manyara, and Kilimanjaro Regions.

### Sampling

Focal villages were selected based on ethnic composition, distance to urban centers and conservation areas, and in consultation with local research assistants. When possible we sampled highland, midland, and lowland environments. This strategy allowed us to investigate AM use by ethnic group, subsistence practice, and geography. Households were randomly selected from census data provided by local village offices (Table 1). Within-households we interviewed a person/persons who knew the most about the household, usually the household head. Given that men are more often considered “household heads” our sample showed a male bias (63% male informants) and all Maasai respondents were male, which prevented us from including sex in statistical models. We control for the effect of other individual attributes on recall, including age, and education, in our models. Our sample is also biased towards dry season data collection given transportation difficulties in the wet season. Maasai were oversampled because of the diversity of herd size, herd mobility and much greater use of over-the-counter AM usage as indicated from pilot research.

### Analytic Strategy

Given recent attention to statistical practice in social and biomedical science [28], we provide these details of our approach. Given little epidemiologic theory for predicting human AM usage in low-income countries, we use stepwise multiple regression to examine the sociocultural factors that may influence dependence on professional and lay veterinary sectors and thus decisions about antibiotic use. We examine three variables with the potential to impact AMR: (a) self-reported professional veterinary consultation (0 = none, 1 = consultation); and (b) self-reported observation of post-treatment “withdrawal” for animal product consumption (1 = some observance, 0 = no withdrawal) and (c) a scale of both self-reported and observed injectable AM use (0 = none to 9 = intensive use). Self-reported items on the AM use scale included whether the household self-administered AMs and recalled ownership of syringes and needles for intramuscular or subcutaneous injections (Table 2). Observed items included inventoried AMs on hand in the household. PCA indicated two dimensions of AM use characterized as low to moderate use, and high use (Table 2). However, Cronbach’s alpha for all nine items indicated good internal consistency (α = 0.80) as a unidimensional scale. We assumed that the combination of self-reported and direct observation items offered a robust measure with good sensitivity and specificity for AM use, resulting in less complicated interpretation of P-values in regression models [29].

**Table 2:** Items included in PCA analysis.

Item	Comp 1	Comp 2
Do you use antibiotics in food animals?*	0.48	-0.10
Do you have a syringe?*	0.45	-0.19
Do you have needles?*	0.43	-0.19
One AM on hand**	0.46	-0.14
Two AMs on hand**	0.32	0.16
Three AMs on hand**	0.18	0.31
Four AMs on hand**	0.17	0.64
Five AMs on hand**	0.08	0.32
Six AMs on hand**	0.08	0.51
Eigenvalue	3.50	1.44

*self-report

**direct observation

We examine 27 “predictor” variables selected based on cross-cultural differences in livelihood (described in Table A in S1 Appendix). We chose a manageable and parsimonious set of variables for this analysis that were most likely to be measured adequately based on (1) our prior experience with parts of the survey instrument and (2) quality control checks for the present study. The following subsistence and sociocultural dimensions may be associated with reliance on professional and lay sectors and could suggest varying selection pressures for AMR:

**Sectors of veterinary care** included visiting a vet drug shop use (0 = no, 1 = yes) or reporting the use of a traditional healer for animals (0 = no, 1 = yes);

**Herd assets and livestock income** were self-reported number of animals managed by the homestead and income derived from animal sales. Herds were separated into three categories for analysis. “Zero-grazing” livestock were those kept in pens within the homestead both day and night. “Daily grazing” livestock were kept in the homestead at night but were free range grazers during the day. “Away grazing” were livestock that were kept away from the household, usually in temporary camps considerable distances from the homestead. For our analysis, we pool sheep and goats together as “small-stock” given similarities in associated herding practices and economic equivalence. Indicators of livestock income/assets included the number of small-stock and cattle sold in the last year; liters per day of cow milk produced and sold; and beef and small-stock meat consumption (1 = never to 6 = everyday).

**Cropland and agricultural income** included self-reports on the acres of crops planted and the amount of crops sold in kilograms.

**Modernization/acculturation** included distance between the homestead and the nearest urban center (observed), respondent education level (1 = illiterate to 7 = post college), whether the homestead had electricity, had a savings account, or owned a bike/motorbike/car/truck, cell phone, or radio (0 = no, 1 = yes).

**Seasonality** included whether the interview occurred during the dry season (June through October) or wet season (November to May) (1 = Dry Season).

**Ethnicity** was coded as 0 = Arusha and 1 = Maasai.

We examine our three outcome variables in multiple regression analyses with Poisson (AM use scale) or Logit (veterinarian consultation, and withdrawal) link functions. Following analysis of a “full” multiple regression model including all predictors and combining ethnic groups, we present models by ethnic group (i.e., Maasai and Arusha) with parsimonious and transparent mechanical model selection that retain variables with significance of 0.1 or below. (see S1 Appendix for model diagnostics). For “full models” the ratio of sample size to predictors (n/k) for AM use was 11.65, which could indicate model instability [30]. See Tables I-K in S1 Appendix for full model results. Using stepwise variable selection (P = 0.09 for entry and 0.10 for removal), we assessed reduced models for AM use with combined ethnic groups resulting in n/k = 51. We examined interactions with ethnicity using separate models for ethnic groups because combined models including interaction effects would lead to unacceptably high multicollinearity and low n/k. For culture group-specific models we used stepwise variable selection as described above but limited to subsamples of 200 Maasai and 91 Arusha households achieved n/k = 100 and 15 respectively for AM use. We exclude Chagga people from multivariate analyses because few livestock owners reported using anything other than professional veterinary care, and almost all livestock owners reported observing withdrawal periods for meat and milk consumption. Consequently, there was little variance to explore among Chagga people (described below). Prior to discussion of model results, we provide an overview of the qualitative and quantitative characteristics of the study populations.

## Results and Discussion

### The Chagga

Chagga People of Mt. Kilimanjaro are consumers of professional veterinary services. Chagga farmers have lived on Mt. Kilimanjaro since the 12^th^-13^th^ centuries [31]. Traditionally, Chagga spoke several dialects of *Kichaga*, a common Bantu language, but dialects are not always mutually intelligible and today most speak *Kiswahili* exclusively. Chagga communities are relatively densely packed on the slopes of Kilimanjaro. A system of highways rings the base of Kilimanjaro, and communities have relatively easy access to markets, government offices, clinics, schools, religious institutions, and pharmacies. Many Chagga still rely on mixed-species gardens, free-range poultry, and small herds of goats, sheep, pigs, and cattle for subsistence and market sales. Other Chagga are fully integrated into 21^st^ century commerce [32]. Higher levels of urbanization, relative prosperity, and early adoption of Christianity have meant that many Chagga have long had access to “Western” education and healthcare [31].

Chagga animal husbandry includes almost exclusive use of certified veterinary professionals for the care of poultry, small-stock, and cattle. Chagga occasionally graze animals in pastures on the slopes of Mt. Kilimanjaro, but most herds are zero-grazing and live in pens in family compounds where people bring fodder and water. These herds consist of an average of four small-stock, and 1.5 cattle (Table B in S1 Appendix). Zero-grazing seems to be “integrated” with seeking professional veterinary care for livestock. Only one Chagga reported administering antibiotics for food animals and another reported reliance on a traditional healer. Almost all households (96%) reported compliance with withdrawal periods before consumption of meat and milk (Table 3). Likely compliance with recommended AM practices and reliance on professional veterinarians suggests that AM use is a less salient risk factor for selection of AMR in Chagga communities compared with more livestock dependent Maasai and Arusha (Table 3).

**Table 3:** Proportion antibiotic use and sectors of care among livestock owners*.

Variable	Chagga	Maasai	Arusha
Certified veterinarian	0.96	0.36	0.45
Veterinary drug shop	0.04	0.58	0.37
Traditional healers	0.01	0.19	0.03
No withdrawal	0.04	0.93	0.28
Self-report lay use of veterinary antibiotics	0.01	0.74	0.21
Owns syringe for veterinary antibiotics	0.02	0.95	0.41

*6% of Chagga, 3% of Maasai, and 18% of Arusha reported no livestock.

### The Maasai

Maasai People of the Maasai Steppe make extensive use of AMs. Maasai and related Maa-speaking pastoralists are found throughout Tanzania and Kenya [33–35]. Traditionally Maasai were nomadic pastoralists who moved with their herds in search of grass and water. Today most Maasai are agro-pastoralists who grow some crops (mostly maize and beans) and keep cattle, sheep and goats, and free-range poultry [33]. Our sample includes populations in Monduli, Ngorongoro, and Simanjiro Districts (Table 1) where Maasai live along an economic continuum from subsistence-level herding and agriculture to intensive cash cropping. Patrilocal, extended family homesteads or *enkang* are the units of production and function as corrals for livestock when animals are not traveling with *moran* (“warriors”) in search of water and grass.

Compared to other ethnic groups, Maasai herds are very large (Table B in S1 Appendix). There appears to be substantial economic inequality and local variation among Maasai herds [36] with 8% of our Maasai sample reporting combined herds of more than 1,000 animals. Men own livestock individually, but herds are pooled for management purposes in the *enkang* where fathers, adult sons, adult brothers, their wives, and children work to manage the stock cooperatively. Combining cattle and small-stock the mean herd managed by an *enkang* was 345 animals with a median of 160. These means are high compared to published data for other Maasai samples in nearby populations. For example, the mean “Tropical Livestock Units” (TLU) was 128 in the nearby village of Sukuro while in our sample the equivalent mean was 140 TLUs (based on methodology reported by Leslie & McCabe [36]). This difference is likely due to different sampling methods: We pooled herds at the smallest unit recorded in village offices, which also happens to be the local Maasai “unit” for herd management and locus of livestock control. Despite individual ownership, patrilocal elders (usually fathers and elder brothers) have considerable influence over general herd management and livestock offtake for meat and sales. Occasionally, intra-*enkang* conflicts over livestock management can lead to *enkang* fission.

Among Maasai, cattle are particularly valued, but small-stock are still important [37]. Cattle provide milk for daily consumption and meat for occasional ritual meals. Like other East African pastoralists, the Maasai are reluctant to sell cattle even in times when need for cash is acute [38]. In contrast, small-stock are a source of meat for regular household consumption, and they may be sold for cash, while consumption of small-stock dairy products was much rarer in our sample (Table A in S1 Appendix). Maasai herds traditionally focused on cattle, but research over the past 40 years has indicated a growing reliance on small-stock, which may be associated with climate change and development [39–41]. Small-stock composed approximately 57% of Maasai herds in our sample on a per head basis. Poultry are much less important than cattle and small-stock: More traditional Maasai typically indicated that they did not eat eggs or poultry, although more acculturated Maasai did. However, it is likely that keeping chickens has increased recently, especially among women as poultry sales comprise a potentially important part of their personal income. In general, the value of different species may vary by *enkang* demographics and may reflect alternative livestock livelihood strategies [36].

Large herds demand substantial time, attention, water, and food to manage. Maasai herds are divided into two groups. The “daily grazing” herd includes animals that sleep in the *enkang* at night, and forage for food and water with boy caretakers during most of the daylight hours. These animals include milk cows for household dairy production that usually produce 0.5 to 1 liter per day for household offtake per lactating animal. The “away grazing” herd includes animals kept in distant cattle camps called *manyata*, where unmarried *moran* manage them while searching for grazing areas and water. *Manyata* may be as close as 8 km from the *enkang* to over 150 km away, depending on local rainfall. The ratio of these two herd components appears to fluctuate seasonally. During the dry season about 68% of animals slept in the *enkang* at night (32% were in a *manyata*), but that proportion increased to 86% in the rainy season with 14% kept in distant *manyata*.

Despite or because of intentionally pursuing a traditional livestock-based livelihoods (see McCabe *et al*. [32]), Maasai people are enthusiastic adopters of new technology for livestock management. Vaccinations, antibiotics, antiprotazoals, antihelminthics, insecticide dips, and sprays are chief among those technological advances. A majority of Maasai households had oxytetracycline (OTC) on hand (80%) while fewer households had penicillin and streptomycin (4%), tylosin (5%), and sulfonimides (4%).

A majority of Maasai (67%) also reported that their cattle were vaccinated against one or more illnesses, and 29% reported that their small-stock were vaccinated (Table D-E in S1 Appendix). Seventy-four percent of livestock owners reported lay AM use. Informants maintained this use was mostly confined to cattle, while veterinary medicines were used sparingly for small-stock and almost never in the care of poultry. Thirty-six percent reported consultation with certified veterinarians (Table 3) although this may be underestimated because livestock extension officers administer vaccinations. Most Maasai (93%) reported consuming meat and milk from animals that were being treated with antibiotics. That is, they observed *no* withdrawal. With widespread AM use, low levels of professional consultation, and exposure to meat and milk from animals under antibiotic treatment, we suggest that the Maasai are more reliant on lay sector veterinary care. As such, AMR selection pressures associated with AM-use behavior are likely to be higher among Maasai compared with other ethnic groups.

Qualitative interviews and quantitative data indicated that AMs are well integrated into Maasai ethnoveterinary practice. The most common illnesses reported (Table 4) are often treated with AMs although most of them are not caused by bacterial pathogens. Tick-borne diseases such as East Coast Fever “ECF” (*Theileria* spp., protozoa), heartwater disease (*Ehrlichia ruminantium*, protozoa) and anaplasmosis (*Anaplasma* spp., bacteria) can be treated to some extent with OTC, while AMs are also frequently used to prevent secondary bacterial infections for animals with Foot and Mouth Disease (picornavirus). Apparently, herd owners have a good idea of which clinical signs call for AM treatment despite having no knowledge of (or interest in) germ theory. Maasai reported that some traditional herbal remedies are as effective as “Swahili” or foreign medicine; nevertheless, they prefer to use foreign medicines because these are easier to obtain than traditional plant medicines that must be foraged from the environment when needed [22]. Among our Maasai sample 68% had at least one AM on hand at the time of the interview, and 94% had a syringe for AM injections in livestock. Note that the proportion of self-reported AM use (Table 3) is lower than reported syringe ownership, indicating either that syringes are kept on hand for professional vets to use during visits or self-reported AM use underestimates actual use. Fewer Maasai livestock owners (34%) reported using over-the-counter AMs in addition to professional veterinary consultation. Antibiotics are generally stored in plastic bags and are probably used and/or replaced quickly because only nine out of the 251 antibiotics examined were beyond their expiration date.

**Table 4:** Common livestock illnesses and frequency of antibiotic use for these presumptive diseases in Maasai households.

Livestock illness	Frequency	Proportion	Treatment
"*Endigana"* (East Coast Fever & Anaplasmosis)	77	0.38	Antibiotic
Pneumonia (CBPP or CCPP^a^, possibly Pasteurellaceae)	51	0.25	Antibiotic
Foot and Mouth Disease (viral)	33	0.16	Antibiotic
Heartwater disease (*Ehrlichia ruminantium*)	20	0.10	Antibiotic
Trypanosomiasis (protozoa)	11	0.05	Antiprotazoal
Anthrax (*Bacillus anthracis*)	9	0.04	None^b^
Malignant Catarrhal Fever (viral)	3	0.01	None
Total	204		

^a^CBPP = contagious bovine pleuropneumonia; CCPP = contagious caprine pleuropneumonia

^b^Typically death is one of the first clinical signs of anthrax.

Key informant interviews about the causes of livestock illness suggest that etiology is not a focus of Maasai attention, which may help explain why AM’s are used for most diseases. A majority of livestock owners in our sample reported that they did not know the specific cause of illnesses, with the exception of trypanosomiasis, whose local name (*Ndorobo*) is the *Kiswahili* word for “tsetse fly”. Some suspected that grazing on toxic grasses could also be important, particularly for the transmission of ECF (*endigana kali* in Maa), which is caused by a group of protozoa (*Theileria spp*.) that are transmitted to livestock through ticks [42]. A Maasai livestock extension officer indicated that he told many livestock owners that ticks transmit ECF, but only some are beginning to believe it. A study by Kioko *et al*. [43] among Maasai in Monduli District (where we also worked) found that 56% of Maasai associated ticks with ECF. Although Maasai are very interested in foreign veterinary medicines, they are much less interested in foreign explanatory models. Instead, animals that die from illness are dissected and observed for gross organ involvement; hence, names like “heart water” etc.

When clinical signs are consistent with an illness known to be sensitive to AMs, the herd owner begins treatment, usually with injectable OTC. If an animal does not respond to treatment within three days, then the owner may increase the dosage, switch to a higher concentration (e.g. from 10% to 20% formulation) or try a different treatment. Herd owners are generally aware of recommended courses of treatment and dosage practices, despite a 59% illiteracy rate among *enkang* heads. For 10% OTC, livestock owners routinely reported giving the animal injections once per day for at least three days (a reasonable course for many infections). Dosage remains problematic because scales are unavailable and it can be difficult to accurately estimate an animal’s weight.

A majority of our Maasai sample treat their animals themselves. Approximately 36% of Maasai did, however, employ the services of a certified veterinarian who is almost always a government livestock extension officer (LEO) (Table 3). These services are sometimes employed in addition to lay AM use. Extension officers are knowledgeable and skilled, but their practice among the Maasai may include over 10,000 animals dispersed over vast and remote territory for a single LEO. Extension officers help with diagnosis, usually based on clinical signs that may be described over a cell phone, although occasionally diagnostic samples are sent to regional laboratories for analysis. One main function of LEOs is to deliver and administer vaccines, which are provided free of charge by the Tanzanian government.

Maasai purchase AMs at special veterinary drug shops that can be found even in the smallest villages. Drug shops are licensed to qualified animal health specialists with certification similar to LEOs. On a day-to-day basis, however, shops may be attended solely by a clerk with no specialized training in veterinary medicine. Livestock owners rarely seek advice from veterinary drug shop attendants. Maasai people report choosing particular AMs after learning about them and their use from friends, family and LEOs. Prices vary considerably by AM, formulation, and location of purchase. A 100 mL vial of 10% OTC costs about $3,750 Tanzania Shillings or $2.50 USD, and it is sufficient to treat about three adult local zebu cattle or 17 adult local variety small-stock for three days. Local veterinary drug shops in more remote areas purchase AMs from retailers in Arusha town. We were only able to identify one outlet in Arusha that purchased AMs directly from wholesalers in Dar es Salaam and Nairobi. Apparently all other local vendors obtained AMs from this one distributor in Arusha. AMs originate from multiple countries under various brand names. China, UK, Ireland and Netherlands were the most common countries of origin for livestock AMs.

Key informants also indicated “brand loyalty” for AMs. LEOs expressed concern that some AM brands are less effective than others although there are no published data addressing the quality of these drugs. Because 59% of *enkang* heads are illiterate, the style of the AM bottle and packaging helps ensure that the correct AM was purchased for its intended use, possibly contributing to brand loyalty. Given the communal nature of *enkang* and secular trends toward universal primary education, nearly all homesteads include at least one literate person who can help identify dosage from AM package instructions.

Compared to veterinary medicine, Maasai appear universally to use certified medical professionals for human healthcare. About 50% of our sample (Table 5) treated the most recent human illness with an unknown “exotic” medication prescribed by a certified medical professional. In contrast, Maasai know the name and use of every “exotic” veterinary medicine in their pharmacopeia. Noteworthy, Maasai reported about twice as much antibiotic use for the most recent human illness compared to Chagga. Arusha are intermediate (Table 6). We cannot be certain to what extent antibiotics are included in “unknown” treatments in Table 6.

**Table 5:** Common antimicrobials in Maasai and Arusha homesteads for veterinary care by proportion of homesteads in the sample who had a particular antibiotic on hand.

Veterinary Antibiotics	Maasai	Arusha
Oxytetracycline (OTC)	0.80	0.15
Penicillin & Streptomycin (Penstrep)	0.04	0.01
Tylosin	0.05	0.07
Sulfonimides	0.02	0.00

**Table 6:** Most recent reported human treatments by household*.

Most recent human treatment	All (n = 391)	Maasai (n = 200)	Chagga (n = 100)	Arusha (n = 91)
None	0.17	0.17	0.19	0.17
Unknown name	0.49	0.47	0.58	0.45
Non-antibiotic medications	0.24	0.25	0.18	0.30
Amoxicillin	0.06	0.07	0.02	0.09
Ampicillin	0.01	0.02	0.01	0.00
Tetracycline	0.01	0.01	0.00	0.00
Other antibiotic (not specified)	0.02	0.03	0.02	0.00
Total antibiotics combined from above	0.093	0.118	0.050	0.088
*P*-value, difference between proportions test; Maasai reference			0.029	0.068

* Data from individuals from other ethnic groups (n = 24) not included.

### The Arusha

The Arusha People, who live on Mt. Meru and on the plains around Arusha Town, exhibit transitional veterinary practices. Arusha AM use reflects similarities with Maasai and Chagga. Arusha share a language and deep cultural history with the Maasai [44]. Like the Chagga, Arusha mostly inhabit peri-urban and midland/highland environments and were also early adopters of colonial influences. Arusha are most likely descendants of either Loogolala or Parakuyo Maasai sections, who were displaced by Kisongo Maasai in the early 19^th^ century [44]. Indeed, Arusha maintain a cultural identity with Maasai; they speak *Maa*, observe Maasai circumcision rituals, consult *laibons*, are inducted into Maasai age-sets, and historically Arusha *moran* raided for cattle and women [45]. During the mid-19^th^ century, Arusha subsistence shifted away from livestock towards cultivation, and began to resemble Chagga subsistence patterns as they moved up the southwestern slopes of Mt. Meru [44]. On Mt. Meru they cultivated beans, bananas, and maize while keeping flocks of free-range poultry and small herds of cattle and small-stock, as the majority of Arusha continue to do today. From Mt. Meru, Arusha exchanged produce with Maasai, particularly during the late 19^th^ century when periods of drought and disease ravaged the Maasai Steppe (e.g., rinderpest). Access to mountain streams, along with small and isolated livestock herds, meant Arusha were less vulnerable to diseases than their Maasai counterparts. With these advantages, Arusha displaced populations inhabiting the plains around present day city of Arusha and began to expand their herds. [45]. Arusha, like Chagga, integrated into regional market economies through the adoption of cash crops, particularly coffee. Early “acculturation” allowed Arusha, unlike most Maasai, access to “Western” healthcare services, including veterinarian medicine [45].

On average, Arusha herds are small compared with Maasai, but larger than Chagga herds (Table B in S1 Appendix). The mean zero-grazing herd for Arusha was 1.1 small-stock and 0.8 cattle. Free-range stock are more common compared to Chagga. Arusha daily grazing herds averaged 5.7 small-stock and 1.6 cattle. Away grazing herds had a mean of 3.6 small-stock and 4.6 cattle.

Across three Arusha villages AM practices shared affinities with both Maasai and Chagga. Over 76% of Arusha households on Mt. Meru indicated reliance upon veterinarian services while only about 10% of those in the two Arusha lowland villages reported using veterinarian services. Cultural continuity with the Maasai may help explain why 24% of Arusha households on Mt. Meru still rely on lay AM use, even though their subsistence patterns, access to modern veterinary services, and distance to urban areas are more similar to Chagga. Other indicators of AM use fall between those reported by Maasai and Chagga: 45% of Arusha reported using government certified veterinarians; 37% used a veterinary drug shop to purchase AMs; 28% of Arusha reported observing no withdrawal for consuming milk and meat (Table 3). A minority of Arusha households had OTC (15%), penicillin and streptomycin (1%), and tylosin (7%) on hand. Like Maasai, Arusha do not use AMs to treat free-range poultry. However, commercial poultry feed is available in Arusha Town, and farmers raising commercial breeds of layers and broilers likely use some antibiotics [46,47].

### Correlation and Regression Analysis

In general, multivariate results indicated that patterns of AM use, vet consultation, and withdrawal were associated with different herd management approaches, livestock species, sectors of vet care, market integration, “acculturation”, and seasonality.

Recognized veterinary standard practices [9] suggest that lay AM use, vet consultation, and observance of withdrawal should be highly correlated. In this sample, however, vet consultation was not correlated with AM (*r* = -0.00) nor withdrawal (*r* = -0.01); and AM use was substantially negatively correlated with withdrawal (*r* = −0.62, S6 Table) indicating that lay AM users tended not to observe recommended withdrawal after treatment. These results suggest that better public health communication through professional vets could reduce AM resistance and exposure to AM residues in food.

Similar to Chagga, zero-grazing was negatively associated with lay AM use, and positively associated with professional vet consultation in models combining Maasai and Arusha data (Table 7). Effects for zero-grazing on outcomes appeared to be culture-specific: Zero-grazing was associated with a greater probability of vet consultation among Arusha, and greater probability of withdrawal among Maasai (Table 8). These results suggest that professional vet care is most accessible in more densely populated, peri-urban areas where zero-grazing is most common. Similarly, among Arusha distance from an urban center was also associated with lower probability of vet consultation (Table 8). Interestingly, the Maasai were more likely to consult a vet than the Arusha after controlling for included variables indicating that underlying socio-economic differences only account for part of the variance in vet consulting behavior. Considering both qualitative and multivariate results, however, recommended veterinary practices appeared to be relatively well integrated with zero-grazing livestock management.

**Table 7:** Reduced models for multiple regression showing significant effects on AM use, vet consultation (VC), and observance of withdrawal (OW) for Maasai and Arusha combined. VC and OW coefficients are presented as logits with positive values indicating log-odd increases in reporting of a behavior and negative indicating a decrease in log-odds.

	AM	VC	OW
Variable	β (P values)	β (P values)	β (P values)
Maasai	1.034(0.000)	1.804(0.002)	-2.546(0.000)
Vet drug shop		-3.366(0.001)	
Traditional Healer		-2.996(0.005)	
Small-stock daily grazing	0.001(0.002)		-0.009(0.012)
Cattle daily grazing			
Small-stock zero grazing			
Cattle zero grazing	-0.232(0.003)	1.198(0.006)	
Small-stock away grazing			-0.017(0.049)
Cattle away grazing			
Small-stock sold last year		-0.117(0.000)	
Cattle sold last year		0.143(0.016)	0.151(0.003)
Cow milk produced lt/day		0.032(0.003)	
Cow milk sold lt/day			
Frequency beef consumption			0.261(0.065)
Frequency small-stock consumption		-0.113(0.082)	
Acres planted in crops			
Kg of crops sold			
Dry season	-0.161(0.014)		-0.751(0.077)
Distance to urban center (km)			
Age (years)			
Education (0 = illiterate, 7 = post college)	0.057(0.054)		
Owns bike, motorbike or car/truck (1 = yes, 0 = no)	0.221(0.067)		
House has electricity			1.086(0.039)
Owns cellphone			
Owns radio			
Has savings account		-0.870(0.045)	
Constant	0.221	-0.113	0.582
Pseudo R2	0.17	0.41	0.47
Prob>chi2	0.00	0.00	0.00
Log Likelihood	-502.87	-115.65	-94.32
LR chi2	209.36	157.46	164.66
Obs	291	291	291

**Table 8:** Reduced models for multiple regression showing significant culture-specific effects on AM use, vet consultation (VC), and observance of withdrawal (OW) by Maasai (bold) and *Arusha (italic)*. VC and OW coefficients are presented as logits with positive values indicating log-odd increases in reporting of a behavior and negative indicating a decrease in log-odds.

	AM	VC	OW
variable	β (P values)	β (P values)	β (P values)
Vet drug shop		**-3**.**621(0**.**000)**	
Traditional Healer		**-2**.**902(0**.**000)**	
Small-stock daily grazing	**0**.**001(0**.**000)**/*0*.*041(0*.*000)*		
Cattle daily grazing	*0*.*100(0*.*001)*	*0*.*227(0*.*042)*	
Small-stock zero grazing			
Cattle zero grazing		*1*.*113(0*.*002)*	**0**.**626(0**.**032)**
Small-stock away grazing	*0*.*025(0*.*002)*		*-0*.*038(0*.*047)*
Cattle away grazing			
Small-stock sold last year		**-0**.**105(0**.**002)**	
Cattle sold last year		**0**.**138(0**.**016)**	
Cow milk produced lt/day		**0**.**022(0**.**039)**	
Frequency beef consumption			
Frequency small-stock consumption		**0**.**493(0**.**013)**	
Acres planted in crops			
Kg of crops sold			
Dry season	**-0**.**147(0**.**073)**	*-1*.*564(0*.*039)*	*-1*.*338(0*.*011)*
Distance to urban center (km)		*-0*.*954(0*.*000)*	
Age (years)		*-0*.*045(0*.*065)*	
Education	*0*.*182(0*.*017)*		
Owns bike, motorbike or vehicle	*0*.*949(0*.*000)*		
House has electricity		**-1**.**410(0**.**055)**	**1**.**398(0**.**035)**
Owns cellphone	*0*.*975(0*.*023)*	**1**.**796(0**.**065)**	
Owns radio		*1*.*408(0*.*088)*	**1**.**931(0**.**014)**
Has savings account			
Constant	**1**.**268**/-*2*.*494*	**-0**.**781**/*7*.*361*	**-4**.**070**/*1*.*933*
Pseudo R2	**0**.**030**/*0*.*320*	**0**.**37/***0*.*51*	**0**.**20**/*0*.*10*
Prob>chi2	**0**.**000**/*0*.*000*	**0**.**000**/*0*.*000*	**0**.**000**/*0*.*000*
Log Likelihood	**-362**.**36**/-*102*.*55*	**-83**.**62**/*-30*.*35*	**-46**.**72**/*-46*.*13*
LR chi2	**21**.**6**/*98*.*1*	**96**.**34**/*62*.*97*	**22**.**88**/*10*.*63*
N	**200**/*91*	**200**/*91*	**200**/*91*

Among the Maasai, three sectors of veterinary care (lay AM use, professional care, and shamanic practice) appeared to be alternatives rather than complementary systems. Maasai who reported purchasing over-the-counter medicines from a veterinary drug shop, and those reporting use of traditional healers were significantly less likely to consult vets for livestock care (Table 8).

Modernization or acculturation variables showed different effects among Maasai and Arusha. Among Arusha owning transportation or cell phone, and education were positively associated with lay AM use (Table 8). Maasai who owned a radio or who had electricity were more likely to observe post-treatment withdrawal (Table 8) suggesting potential for public health communication via local radio stations.

Cattle production for market sales and dairy production were associated with greater probability of vet consultation (Table F in S1 Appendix) and these associations were specific to Maasai (Table 8). Sale of small-stock, in contrast, was associated with lower probability of professional vet consultation (Table 8). This is consistent with different roles for cattle and small-stock in Maasai production systems [37,39].

Results for species composition of herds are difficult to interpret. Size of the daily grazing small-stock herd was positively associated with AM use for both Maasai and Arusha (Table 7). For Arusha both cattle and small-stock grazing herds were positively associated with AM and cattle were positively associated with vet consultation (Table 8). Growing emphasis on small-stock production among African pastoralists over recent decades has been interpreted as a response to market integration and drying climate [36, 38–40]. Among East African pastoralists small-stock are important for cash sales and daily meat while cattle are important for daily milk consumption and meat for special rituals [37,39]. These patterns of changing herd composition may significantly impact AM use and suggest avenues for future research. Given that AMs are rarely used to treat small-stock in the Maasai or the Arusha, this association may derive from the economic contributions of selling small-stock. Because small-stock are more likely to be sold, those with larger herds may simply have more regular inputs of cash to purchase AMs (to treat cattle). However, functional relationships between within-group livestock management and participation in either professional or lay veterinary sectors are unclear.

Seasons in Tanzania are generally characterized as wet and dry, with some local variation in timing. The dry season was negatively associated with AM use among Maasai, but not Arusha; however, with *P* = 0.07 the association did not reach conventional significance (Table 8). Arusha were less likely to consult vets or observe recommended withdrawal during the dry season (Table 8). As noted above, daily grazing livestock are a smaller proportion of herds in the dry season when animals travel to distant livestock camps in search of forage and water. Hence, household milk production is reduced in the dry season making withdrawal of milk potentially more costly for household nutrition. Fewer animals in the homestead during the dry season might also influence motivation for vet consultation.

## Caveats and Conclusions

Across northern Tanzania, dependence on professional and lay sectors of veterinary care vary with ethnic differences in herd management and composition, market sales, acculturation and geography. The exploratory analytical approach was motivated by a lack of predictive theory in anthropology and epidemiology for veterinary decisions among livestock owners. Economic models of risk perception and motivations for medical treatment may prove to be particularly useful. A parallel economic analysis of AM use in this sample using an “expected profit maximization” framework indicated that prior illness experience, livestock exposure, distance to markets, and household income influenced decisions to use AMs for livestock management [48]. Those results based on two-stage regression suggest that effects of small-stock on AM observed in our analysis could be mediated by monetized income measures. Such mediation is consistent with our interpretation that small-stock can be transformed into cash used to purchase antibiotics. Future research might benefit from close integration of economic models with ethnographic data.

A multilevel, multi-factorial approach will likely improve predictive models of AMR. At one level of analysis a zero-grazing “neighborhood” on the slopes of Mt. Meru or Kilimanjaro or a shared Maasai rangeland represents a local environment for selection of resistance. On another level, livestock, people, bacteria, ticks, meat, and milk travel beyond local boundaries. Relatively close contact among culture groups including large mobile herds– 72,000 animals in our small Maasai sample—with high levels of AM use, low levels of professional consultation, and absence of withdrawal suggests potentially important regional effects for dissemination of resistance depending on contact among people, animals, and bacteria across communities.

An important caveat to these conclusions is that self-reported data on AM-use practices may suffer from social desirability bias, particularly if the respondents are aware of risks associated with AM use and lack of withdrawal. We might generally expect underreported AM use, but we expect this bias is attenuated in less literate populations. Future research could combine self-report, experimental, and observational data to determine the degree and influence of response bias in AMR studies. More reliable and efficient behavioral science methods for estimating rates of use, dosage, etc. would be very useful for estimating potential antibiotic selection pressures and relationships with sociocultural factors. Likewise, careful attention to and measurement of professional veterinarian practices could identify additional selection pressures that we cannot address in the present study.

While our results suggest that better public health communication between the professional and lay veterinary sectors could help mitigate selection for AMR, AM use in livestock represents only one dimension in a multifaceted selection and transmission environment. Factors such as the therapeutic use of AMs in human healthcare, access to clean water and sanitation, disease distribution, and the densities and movements of human and livestock populations, can all impact selection and transmission of AMR [49]. Use of AMs in human healthcare may be particularly concerning in low- and middle-income countries given over-the-counter availability [50]. Improved predictive models of AMR should simultaneously assess AM use in humans and livestock, especially in low-income countries where many people are in close contact with livestock, and where all AMs tend to be less regulated. Such an integrated “one-health” approach will prove necessary to understand and limit the emergence, persistence and transmission of AMR.

## Supporting Information

S1 Appendix Additional information on survey development, survey methods, and analyses. Analysis include correlation matrices for model variables and models including all predictors.
(DOCX)

S2 Appendix Household survey questionnaire. (PDF)

S1 Dataset Dataset used in study analyses. (CSV)

## References

[1] Davies J, Davies D. Origins and Evolution of Antibiotic Resistance. Microbiol Mol Biol Rev. 2010;74: 417–433. 10.1128/MMBR.00016-10 20805405 PMC2937522

[2] Levy SB, Marshall B. Antibacterial resistance worldwide: causes, challenges and responses. Nat Med. 2004;10: S122–S129. 10.1038/nm1145 15577930

[3] Palmer GH, Call DR. Antimicrobial resistance: A global public health challenge requiring a global one health strategy [Internet]. 2013. https://www.iom.edu/~/media/Files/Perspectives-Files/2013/Commentaries/BGH-Anitmicrobial-Resistance.pdf

[4] Silbergeld EK, Graham J, Price LB. Industrial Food Animal Production, Antimicrobial Resistance, and Human Health. Annu Rev Public Health. 2008;29: 151–169. 10.1146/annurev.publhealth.29.020907.090904 18348709

[5] Carlet J, Jarlier V, Harbarth S, Voss A, Goossens H, Pittet D. Ready for a world without antibiotics? The Pensières Antibiotic Resistance Call to Action. Antimicrob Resist Infect Control. 2012;1: 1–13. 22958833 10.1186/2047-2994-1-11 PMC3436635

[6] Landers TF, Cohen B, Wittum TE, Larson EL. A Review of Antibiotic Use in Food Animals: Perspective, Policy, and Potential. Public Health Rep. 2012;127: 4–22. 22298919 10.1177/003335491212700103 PMC3234384

[7] Eltayb A, Barakat S, Marrone G, Shaddad S, Stålsby Lundborg C. Antibiotic Use and Resistance in Animal Farming: A Quantitative and Qualitative Study on Knowledge and Practices among Farmers in Khartoum, Sudan. Zoonoses Public Health. 2012;59: 330–338. 10.1111/j.1863-2378.2012.01458.x 22333519

[8] Sarmah AK, Meyer MT, Boxall ABA. A global perspective on the use, sales, exposure pathways, occurrence, fate and effects of veterinary antibiotics (VAs) in the environment. Chemosphere. 2006;65: 725–759. 10.1016/j.chemosphere.2006.03.026 16677683

[9] Omulo S, Thumbi SM, Njenga MK, Call DR. A review of 40 years of enteric antimicrobial resistance research in Eastern Africa: what can be done better? Antimicrob Resist Infect Control. 2015;4: 1–13. 10.1186/s13756-014-0041-4 25717374 PMC4339253

[10] Redding LE, Cubas-Delgado F, Sammel MD, Smith G, Galligan DT, Levy MZ, et al The use of antibiotics on small dairy farms in rural Peru. Prev Vet Med. 2014;113: 88–95. 10.1016/j.prevetmed.2013.10.012 24188819 PMC4197910

[11] Graeub BE, Chappell MJ, Wittman H, Ledermann S, Kerr RB, Gemmill-Herren B. The State of Family Farms in the World. World Dev.

[12] FAO. World Agriculture: Towards 2015/2030 [Internet]. Food and Agriculture Organization; 2013.

[13] Steinfeld H. The livestock revolution—a global veterinary mission. Divers Prog Vet Parasitol Res 21st Century Sel Present 19th Int Conf World Assoc Adv Vet Parasitol. 2004;125: 19–41. 10.1016/j.vetpar.2004.05.003 15476965

[14] Darwish WS, Eldaly EA, El-Abbasy MT, Ikenaka Y, Nakayama S, Ishizuka M. Antibiotic residues in food: the African scenario. Jpn J Vet Res. 2013;61: S13–S22. 23631148

[15] Roderick S, Stevenson P, Mwendia C, Okech G. The Use of Trypanocides and Antibiotics by Maasai Pastoralists. Trop Anim Health Prod. 2000;32: 361–374. 11147276 10.1023/a:1005277518352

[16] McCorkle CM, Mathias E. Ethnoveterinary medicine in Africa. Africa. 1992;62: 59–93.

[17] Anyinam C. Availability, accessibility, acceptability, and adaptibility: Four attributes of African ethno-medicine. Soc Sci Med. 1987;25: 803–811. 3317889 10.1016/0277-9536(87)90038-4

[18] Sato A. Does socio-economic status explain use of modern and traditional health care services? Soc Sci Med. 2012;75: 1450–1459. 10.1016/j.socscimed.2012.05.032 22818490

[19] Helman CG. Culture, Health and Illness, Fifth edition London: Hodder Arnold; 2007.

[20] Kleinman A. Patients and Healers in the Context of Culture: An Exploration of the Borderland between Anthropology, Medicine, and Psychiatry. Berkeley, CA: University of California Press; 1981.

[21] Fratkin E. Laibon. Lanham, MA: AltaMira Press; 2011.

[22] Roulette C, Njau EF, Quinlan MB, Quinlan RJ, Call DR. Maasai dietary additives in Tanzania: ethnomedical beliefs, ethnopharmacology, and gender differences.

[23] McCorkle CM. Back to the future: Lessons from ethnoveterinary RD&E for studying and applying local knowledge. Agric Hum Values. 1995;12: 52–80.

[24] Bernard HR. Research methods in anthropology: Qualitative and quantitative approaches. Fourth Walnut Creek, Calif: Altamira Press; 2011.

[25] Thumbi SM, Njenga MK, Marsh TL, Noh S, Otiang E, Munyua P, et al Linking Human Health and Livestock Health: A “One-Health” Platform for Integrated Analysis of Human Health, Livestock Health, and Economic Welfare in Livestock Dependent Communities. PLoS ONE. 2015;10: e0120761 10.1371/journal.pone.0120761 25798951 PMC4370696

[26] Quinlan RJ, Quinlan MB, Dira S, Caudell M, Sooge A, Assoma AA. Vulnerability and Resilience of Sidama Enset and Maize Farms in Southwestern Ethiopia. J Ethnobiol. 2015;35: 314–336.

[27] Caudell M, Rotolo T, Grima M. Informal lending networks in rural Ethiopia. Soc Netw. 2015;40: 34–42.

[28] Wasserstein RL, Lazar NA. The ASA’s statement on p-values: context, process, and purpose. Am Stat. 2016; 00–00.

[29] Colquhoun D. An investigation of the false discovery rate and the misinterpretation of p-values. R Soc Open Sci. 2014;1. 10.1098/rsos.140216 PMC4448847 26064558

[30] Babyak MA. What you see may not be what you get: a brief, nontechnical introduction to overfitting in regression-type models. Psychosom Med. 2004;66: 411–421. 15184705 10.1097/01.psy.0000127692.23278.a9

[31] Stahl KM. History of the Chagga people of Kilimanjaro. New York: Mouton; 1964.

[32] Campbell DJ, Misana SB, Olson JM. Comparing the Kenyan and Tanzanian Slopes of Mt. Kilimanjaro: Why are the neighbouring land uses so different? [Internet]. Nairobi, Kenya: Land Use Change, Impact and Dynamics Project (LUCID); 2004 http://www.lucideastafrica.org/publications/Campbell_LUCID_WP_44.pdf

[33] McCabe JT, Leslie PW, DeLuca L. Adopting Cultivation to Remain Pastoralists: The Diversification of Maasai Livelihoods in Northern Tanzania. Hum Ecol. 2010;38: 321–334. 10.1007/s10745-010-9312-8 PMC3170717 21915157

[34] Roth EA, Fratkin E. As Pastoralists Settle: Social, Health, and Economic Consequences of the Pastoral Sedentarization in Marsabit District, Kenya. New York: Kluwer; 2005.

[35] Hodgson DL. Once intrepid warriors: Gender, ethnicity, and the cultural politics of Maasai development. Bloomington: Indiana University Press; 2001.

[36] Leslie P, McCabe JT. Response Diversity and Resilience in Social-Ecological Systems. Curr Anthropol. 2013;54: 114–143. 24855324 10.1086/669563 PMC4028135

[37] Quinlan RJ, Rumas Isaya, Naisikye Godfrey, Quinlan Marsha, Yoder Jonathan. Searching for Symbolic Value of Cattle: Tropical Livestock Units, Market Price and Cultural Value of Maasai Livestock. Washington State University; 2016.

[38] McCabe JT. Cattle bring us to our enemies. Ann Arbor, MI: University of Michigan Press Ann Arbor; 2004.

[39] Fratkin E. Surviving drought and development: Ariaal pastoralists of northern Kenya. Westview Press; 1991.

[40] Fratkin E. East African Pastoralism in Transition: Maasai, Boran, and Rendille Cases. Afr Stud Rev. 2001;44: 1–25.

[41] Galvin KA. Transitions: Pastoralists Living with Change. Annu Rev Anthropol. 2009;38: 185–198.

[42] Norval RAI, Perry BD, Young AS. The Epidemiology of Theileriosis in Africa. London: Academic Press; 1992.

[43] Kioko J, Baker J, Shannon A, Kiffner C. Ethnoecological knowledge of ticks and treatment of tick-borne diseases among Maasai people in Northern Tanzania. Vet World. 2015;8: 755–762. 10.14202/vetworld.2015.755-762 27065643 PMC4825278

[44] Spear T, Nurse D. Maasai farmers: the evolution of Arusha agriculture. Int J Afr Hist Stud. 1992;25: 481–503.

[45] Spear T. Mountain Farmers: Moral Economies of Land and Agricultural Development in Arusha and Meru. Berkeley: University of California Press; 1997.

[46] Rugumisa BT, Call DR, Mwanyika GO, Mrutu RI, Luanda CM, Lyimo BM, et al Prevalence of Antibiotic-Resistant Fecal Escherichia coli Isolates from Penned Broiler and Scavenging Local Chickens in Arusha, Tanzania. J Food Prot. 2016;79: 1424–1429. 10.4315/0362-028X.JFP-15-584 27497131

[47] Rugumisa BT, Call DR, Mwanyika GO, Subbiah M, Buza J. Comparison of the prevalence of antibiotic-resistant Escherichia coli isolates from commercial-layer and free-range chickens in Arusha district, Tanzania. Afr J Microbiol Res. 2016;10: 1422–1429.

[48] Ahmed H, Yoder J, Quinlan RJ. Risk perceptions, management practices, and demand for livestock antibiotics among agropastoralists. [Internet]. Pullman, WA: Washington State University School of Economic Sciences; Report No.: 2016–17. http://ses.wsu.edu/wp-content/uploads/2016/10/WP2016-17.pdf.

[49] Turnidge J, Christiansen K. Antibiotic use and resistance—proving the obvious. The Lancet. 2005;365: 548–549. 10.1016/S0140-6736(05)17920-3 15708081

[50] Morgan DJ, Okeke IN, Laxminarayan R, Perencevich EN, Weisenberg S. Non-prescription antimicrobial use worldwide: a systematic review. Lancet Infect Dis. 2011;11: 692–701. 10.1016/S1473-3099(11)70054-8 21659004 PMC3543997

