# Supplementary material for: Antimicrobial Use and Veterinary Care among Agro-Pastoralists in Northern Tanzania
Source: PLoS One. 2017 Jan 26;12(1):e0170328. doi: 10.1371/journal.pone.0170328 (PMC5268417; doi:10.1371/journal.pone.0170328)
Supplement: S1 Appendix — Analysis include correlation matrices for model variables and models including all predictors. (DOCX) [file pone.0170328.s001.docx]

**Supporting Information**

**Survey Development**

A mixed-methods approach combining informal and formal interviews with direct observations guided survey development. Informal interviews were conducted during the course of direct observation, including conversations that recounted people’s health experiences, those of their livestock, and additional detail on the circumstances surrounding illness events We observed livestock management and veterinary care in multiple Maasai and Chagga households from 2012 to 2015 including use of VAs, chemical dips, and traditional treatments; necropsy of recently deceased animals; slaughter and butchering; milking and milk handling; breeding; birthing; branding; grazing and fodder provision; and castration. We observed and recorded 85 livestock transactions in Terat market, Simanjiro, and conducted 37 short key-informant interviews in 2015 that informed our knowledge of livestock species value and exchange ratios. Iterative qualitative interviewing helped to add or modify existing survey instruments as different ethnic groups were studied and as lab results of fecal and milk samples suggested new items for inclusion.

**Consideration of Herd size**

Given the large herd sizes that are maintained by Maasai households with no written records about herd management, widely disbursed grazing areas, and an estimated 72,000 or more animals within our sampling frame (totaled from Table 2), it was not practical to make direct head counts. Maasai use a base-ten counting system in Maa; however, respondents tended to round self-reported herd sizes to the nearest five for smaller herds and nearest 10 for large herds. This tendency may have introduced error in our analyses. We assumed that this error is randomly distributed and we have no reason to expect otherwise.

**Table A. Summary statistics.** For variable definitions see “Methods and Materials” in manuscript.

| **Variable** |  | **N** | **Mean** | **SD** | **Min** | **Max** |
| --- | --- | --- | --- | --- | --- | --- |
| Ethnic group | Maasai (1=yes, 0=no) | 425 | 0.49 | 0.50 | 0 | 1 |
|  | Arusha | 425 | 0.21 | 0.41 | 0 | 1 |
|  | Chagga | 425 | 0.24 | 0.43 | 0 | 1 |
| Season | Dry season (1=dry, 0=rainy) | 407 | 0.69 | 0.46 | 0 | 1 |
| Antibiotics use | Antibiotic use | 414 | 2.07 | 2.02 | 0 | 7 |
|  | Eat meat/milk from treated animal | 425 | 0.52 | 0.50 | 0 | 1 |
| Sectors of vet care | Veterinarian (1=use, 0=no use) | 425 | 0.50 | 0.50 | 0 | 1 |
|  | Vet drug shop | 425 | 0.37 | 0.48 | 0 | 1 |
|  | Traditional healer | 425 | 0.10 | 0.31 | 0 | 1 |
| Herd composition | Small stock daily grazing | 425 | 78.14 | 161.94 | 0 | 1200 |
|  | Cattle daily grazing | 425 | 48.12 | 191.54 | 0 | 3150 |
|  | Small stock zero grazing | 425 | 1.32 | 2.84 | 0 | 21 |
|  | Cattle zero grazing | 425 | 0.59 | 1.07 | 0 | 5 |
|  | Small stock away grazing | 425 | 21.69 | 111.43 | 0 | 1810 |
|  | Cattle away grazing | 425 | 27.21 | 112.69 | 0 | 1500 |
| Livestock production | Small stock sold last year | 425 | 3.75 | 10.10 | 0 | 100 |
|  | Cattle sold last year | 425 | 1.57 | 4.49 | 0 | 50 |
|  | Cow milk produced lt/day | 425 | 6.17 | 14.03 | 0 | 100 |
|  | Beef consumption (0=never, 6=daily) | 415 | 2.53 | 1.25 | 0 | 6 |
|  | Small-stock consumption | 415 | 1.78 | 1.23 | 0 | 6 |
| Agriculture | Acres planted in crops | 425 | 9.99 | 21.05 | 0 | 300 |
|  | Kg of crops sold | 425 | 8.80 | 23.27 | 0 | 303 |
| Modernization | Distance to urban center (km) | 408 | 46.27 | 41.23 | 3.59 | 175 |
|  | Age (years) | 415 | 46.54 | 16.75 | 0 | 100 |
|  | Education (0=illiterate, 7=post college) | 416 | 1.04 | 1.15 | 0 | 7 |
|  | House has electricity (1=yes, 0=no) | 416 | 0.17 | 0.38 | 0 | 1 |
|  | Owns bike, motorbike or car/truck | 415 | 0.37 | 0.48 | 0 | 1 |
|  | Owns cellphone | 415 | 0.88 | 0.33 | 0 | 1 |
|  | Owns radio | 416 | 0.59 | 0.49 | 0 | 1 |
|  | Has savings account | 425 | 0.33 | 0.47 | 0 | 1 |

**Table B. Herd composition for Chagga, Maasai, and Arusha households.**

| **Chagga (N=101)** | **Mean** | **SD** | **Min** | **Max** |
| --- | --- | --- | --- | --- |
| Small-stock zero grazing | 3.9 | 3.3 | 0 | 15 |
| Cattle zero grazing | 1.5 | 1.1 | 0 | 4 |
| Small-stock daily grazing | 0.6 | 6.0 | 0 | 60 |
| Cattle daily grazing | 1.2 | 11.9 | 0 | 120 |
| Small-stock away grazing | 1.0 | 10.0 | 0 | 100 |
| Cattle away grazing | 0.8 | 8.0 | 0 | 80 |
| **Maasai (N=210)** |  |  |  |  |
| Small-stock zero grazing | 0.1 | 1.1 | 0 | 15 |
| Cattle zero grazing | 0.1 | 0.5 | 0 | 5 |
| Small-stock daily grazing | 155.1 | 203.4 | 0 | 1200 |
| Cattle daily grazing | 95.9 | 264.2 | 0 | 3150 |
| Small-stock away grazing | 41.6 | 155.8 | 0 | 1810 |
| Cattle away grazing | 52.6 | 155.4 | 0 | 1500 |
| **Arusha (N=91)** |  |  |  |  |
| Small-stock zero grazing | 1.1 | 2.5 | 0 | 13 |
| Cattle zero grazing | 0.8 | 1.3 | 0 | 5 |
| Small-stock daily grazing | 5.7 | 11.4 | 0 | 62 |
| Cattle daily grazing | 1.6 | 3.4 | 0 | 21 |
| Small-stock away grazing | 3.6 | 12.6 | 0 | 80 |
| Cattle away grazing | 4.6 | 25.8 | 0 | 240 |

**Table C. Summary Statistics for Maasai and Arusha households.**

| **Variable** |  | **Maasai** | **Mean** | **SD** | **Min** | **Max** | **Arusha** | **Mean** | **SD** | **Min** | **Max** |
| --- | --- | --- | --- | --- | --- | --- | --- | --- | --- | --- | --- |
| Season | Dry season (1=dry, 0=rainy) | 202 | 0.72 | 0.45 | 0 | 1 | 91 | 0.43 | 0.50 | 0 | 1 |
| Antibiotic use | Antibiotic use | 210 | 3.59 | 1.42 | 0 | 7 | 91 | 1.08 | 1.57 | 0 | 5 |
|  | Eat meat/milk from treated animal | 210 | 0.91 | 0.28 | 0 | 1 | 91 | 0.25 | 0.44 | 0 | 1 |
| Sectors of | Veterinarian (1=use, 0=not use) | 210 | 0.37 | 0.48 | 0 | 1 | 91 | 0.42 | 0.50 | 0 | 1 |
| vet care | Vet drug shop | 210 | 0.57 | 0.50 | 0 | 1 | 91 | 0.33 | 0.47 | 0 | 1 |
|  | Traditional healer | 210 | 0.19 | 0.39 | 0 | 1 | 91 | 0.02 | 0.15 | 0 | 1 |
| Herd | Small stock daily grazing | 210 | 155.10 | 203.36 | 0 | 1200 | 91 | 5.68 | 11.36 | 0 | 62 |
| composition | Cattle daily grazing | 210 | 95.95 | 264.21 | 0 | 3150 | 91 | 1.62 | 3.42 | 0 | 21 |
|  | Small stock zero grazing | 210 | 0.11 | 1.12 | 0 | 15 | 91 | 1.13 | 2.47 | 0 | 13 |
|  | Cattle zero grazing | 210 | 0.08 | 0.55 | 0 | 5 | 91 | 0.85 | 1.27 | 0 | 5 |
|  | Small stock away grazing | 210 | 41.58 | 155.82 | 0 | 1810 | 91 | 3.65 | 12.59 | 0 | 80 |
|  | Cattle away grazing | 210 | 52.56 | 155.44 | 0 | 1500 | 91 | 4.56 | 25.81 | 0 | 240 |
| Livestock | Small stock sold last year | 210 | 6.79 | 13.57 | 0 | 100 | 91 | 1.05 | 2.45 | 0 | 12 |
| Production | Cattle sold last year | 210 | 2.72 | 6.08 | 0 | 50 | 91 | 0.69 | 1.31 | 0 | 9 |
|  | Cow milk produced lt/day | 210 | 11.42 | 17.78 | 0 | 100 | 91 | 1.71 | 7.95 | 0 | 75 |
|  | Beef consumption (0=never, 6=daily) | 210 | 2.04 | 1.10 | 1 | 6 | 91 | 2.81 | 1.44 | 0 | 6 |
|  | Small-stock consumption | 210 | 2.06 | 1.18 | 0 | 6 | 91 | 1.95 | 1.33 | 0.5 | 6 |
| Agriculture | Acres planted in crops | 210 | 14.60 | 28.53 | 0 | 300 | 91 | 4.15 | 4.99 | 0 | 26 |
|  | Kg of crops sold | 210 | 12.29 | 28.66 | 0 | 303 | 91 | 4.71 | 7.27 | 0 | 40 |
| Modernization | Distance to urban center (km) | 203 | 74.48 | 40.58 | 5.4 | 174.9 | 91 | 7.93 | 2.42 | 3.5 | 12.6 |
|  | Age (years) | 209 | 46.47 | 15.59 | 0 | 97 | 91 | 43.51 | 16.14 | 17 | 90 |
|  | Education (0=illiterate, 7=post college) | 210 | 0.60 | 1.09 | 0 | 7 | 91 | 1.25 | 1.17 | 0 | 6 |
|  | Own bike/motorbike/car/truck (1=yes, 0=no) | 209 | 0.36 | 0.48 | 0 | 1 | 91 | 0.53 | 0.50 | 0 | 1 |
|  | House has electricity | 210 | 0.09 | 0.29 | 0 | 1 | 91 | 0.26 | 0.44 | 0 | 1 |
|  | Owns cellphone | 210 | 0.94 | 0.24 | 0 | 1 | 91 | 0.84 | 0.37 | 0 | 1 |
|  | Owns radio | 210 | 0.50 | 0.50 | 0 | 1 | 91 | 0.65 | 0.48 | 0 | 1 |
|  | Has savings account | 210 | 0.23 | 0.42 | 0 | 1 | 91 | 0.35 | 0.48 | 0 | 1 |

**Table D. Vaccination types in Cattle.** Proportion is the proportion of households responding that their cattle had been vaccination against a particular disease.

|  |  | **Maasai** |  | **Chagga** |  | **Arusha** |
| --- | --- | --- | --- | --- | --- | --- |
|  | N | Proportion | N | Proportion | N | Proportion |
| any vaccination | 136 | 0.67 | 49 | 0.51 | 34 | 0.45 |
| ECF^a^ | 92 | 0.45 | 2 | 0.02 | 13 | 0.17 |
| CBPP^b^ | 59 | 0.29 | 4 | 0.04 | 5 | 0.07 |
| anthrax | 39 | 0.19 | 28 | 0.29 | 14 | 0.19 |
| trypanosomiasis | 12 | 0.05 | 0 | 0.00 | 0 | 0.00 |
| FMD^c^ | 11 | 0.05 | 4 | 0.04 | 1 | 0.01 |
| heart water | 6 | 0.03 | 0 | 0.00 | 1 | 0.01 |
| rinderpest | 3 | 0.01 | 0 | 0.00 | 0 | 0.00 |
| anaplasmosis | 3 | 0.01 | 0 | 0.00 | 0 | 0.00 |
| rift valley fever | 1 | 0.00 | 4 | 0.04 | 1 | 0.01 |
| lumpy skin disease | 1 | 0.00 | 15 | 0.16 | 3 | 0.04 |
| N livestock owners | 203 | | 95 | | 75 | |

^a^East Coast fever  ^b^ contagious bovine pleuropneumonia ^c^ Foot and mouth disease

**Table E. Vaccination types in small stock.** Proportion is the proportion of households responding that their small-stock had been vaccination against a particular disease.

| **Small-stock** |  | **Maasai** |  | **Chagga** |  | **Arusha** |
| --- | --- | --- | --- | --- | --- | --- |
|  | N | Proportion | N | Proportion | N | Proportion |
| any vaccination | 59 | 0.29 | 17 | 0.18 | 8 | 0.11 |
| CCPP^a^ | 23 | 0.11 | 3 | 0.03 | 2 | 0.03 |
| PPR^b^ | 15 | 0.07 | 0 | 0.00 | 0 | 0.00 |
| anthrax | 11 | 0.05 | 15 | 0.16 | 5 | 0.07 |
| ECF^c^ | 10 | 0.05 | 0 | 0.00 | 1 | 0.01 |
| rinderpest | 3 | 0.01 | 0 | 0.00 | 0 | 0.00 |
| skin worm | 3 | 0.01 | 0 | 0.00 | 0 | 0.00 |
| emukuji(skin diseases) | 3 | 0.01 | 0 | 0.00 | 0 | 0.00 |
| heartwater | 3 | 0.01 | 0 | 0.00 | 0 | 0.00 |
| unknown | 2 | 0.01 | 0 | 0.00 | 0 | 0.00 |
| N livestock owners | 203 |  | 95 |  | 75 |  |

^a^contagious caprine pleuropneumonia ^b^ peste des petits ruminants ^c^ East Coast fever

**Table F. Pearson’s correlation coefficient for model variables across Maasai and Arusha.**

|  |  | **AM** | **VC** | **OW** |
| --- | --- | --- | --- | --- |
| Antibiotic use | Antibiotic usage | 1.00 |  |  |
|  | Veterinarian (1=use, 0=not use) | 0.00 | 1.00 |  |
|  | Observe withdrawal from meat and milk | -0.62** | -0.01 | 1.00 |
| Sectors of vet care | Vet drug shop | 0.15** | -0.44** | -0.28** |
|  | Traditional healer | 0.21** | -0.13** | -0.20** |
| Ethnicity | Maasai (1=yes, 0=no) | 0.65** | 0.00 | -0.72** |
| Herd composition | Small stock daily grazing | 0.50** | 0.09* | -0.35** |
|  | Cattle daily grazing | 0.25** | 0.15** | -0.18** |
|  | Small stock zero grazing | -0.22** | 0.15** | 0.24** |
|  | Cattle zero grazing | -0.37** | 0.34** | 0.27** |
|  | Small stock away grazing | 0.09* | -0.04 | -0.13** |
| Livestock production | Cattle away grazing | 0.21** | -0.06 | -0.14** |
|  | Small stock sold last year | 0.14** | -0.09* | -0.13** |
|  | Cattle sold last year | 0.15** | 0.05 | -0.08 |
|  | Cow milk sold lt/day | 0.09 | 0.12* | -0.06 |
|  | Cow milk produced lt/day | 0.35** | 0.14** | -0.24** |
|  | Frequency beef consumption (0=never, 6=daily) | -0.18** | 0.03 | 0.28** |
| Agriculture | Frequency small-stock consumption | 0.11* | 0.01 | -0.01 |
|  | Acres planted in crops | 0.21** | 0.01 | -0.19** |
|  | Kg of crops sold | 0.07 | 0.06 | -0.08 |
| Modernization | Distance to urban center (km) | 0.46** | 0.04 | -0.52** |
|  | Age (years) | 0.04 | 0.07 | -0.11* |
|  | Education (0=illiterate, 7=post college) | -0.12** | 0.04 | 0.25** |
|  | Owns bike, motorbike or car/truck (1=yes, 0=no) | 0.02 | 0.00 | 0.14** |
|  | House has electricity | -0.16** | -0.02 | 0.26** |
|  | Owns cell phone | 0.17** | 0.01 | -0.15** |
|  | Owns radio | -0.04 | 0.12** | 0.17** |
|  | Has saving account | -0.07 | -0.02 | 0.08 |
| Season | Dry season | 0.01 | -0.06 | -0.18** |

**Table G. Pearson’s correlation coefficient among model variables for Maasai.**

|  |  | **AM** | **VC** | **OW** |
| --- | --- | --- | --- | --- |
| Antibiotic use | Antibiotic usage | 1.00 |  |  |
|  | Veterinarian (1=use, 0=not use) | 0.15** | 1.00 |  |
| Sectors of vet care | Observe withdrawal from milk and meat | -0.32** | -0.02 | 1.00 |
|  | Vet drug shop | -0.08 | -0.46** | -0.14** |
|  | Traditional healer | 0.16** | -0.14** | -0.10 |
| Herd composition | Small stock daily grazing | 0.42** | 0.14* | -0.13 |
|  | Cattle daily grazing | 0.20** | 0.19** | -0.07 |
|  | Small stock zero grazing | -0.02 | 0.07 | 0.09 |
|  | Cattle zero grazing | -0.14** | 0.16** | 0.30** |
|  | Small stock away grazing | -0.01 | -0.05 | -0.04 |
|  | Cattle away grazing | 0.15** | -0.08 | -0.01 |
| Livestock production | Small stock sold last year | -0.05 | -0.11 | 0.11 |
|  | Cattle sold last year | 0.03 | 0.05 | 0.16** |
|  | Cow milk sold lt/day | 0.08 | 0.12** | 0.00 |
|  | Cow milk produced lt/day | 0.25** | 0.16** | -0.05 |
|  | Frequency beef consumption (0=never, 6=daily) | -0.03 | 0.01 | 0.10 |
|  | Frequency small-stock consumption | 0.12* | 0.06 | 0.05 |
| Agriculture | Acres planted in crops | 0.11 | 0.01 | -0.06 |
|  | Kg of crops sold | 0.04 | 0.07 | 0.00 |
| Modernization | Distance to urban center (km) | 0.04 | 0.12* | -0.13* |
|  | Age (years) | -0.03 | 0.05 | 0.01 |
|  | Education (0=illiterate, 7=post college) | 0.04 | 0.10 | 0.11 |
|  | Owns bike, motorbike or car/truck (1=yes, 0=no) | 0.11 | 0.04 | 0.12* |
|  | House has electricity | 0.02 | -0.07 | 0.20** |
|  | Owns cell phone | 0.05 | 0.07 | -0.06 |
|  | Owns radio | 0.09 | 0.12* | 0.24** |
|  | Has saving account | 0.02 | -0.03 | 0.15** |
| Season | Dry season | -0.27** | 0.01 | 0.12* |

*P ≤0.10; **P≤0.05

**Table H. Pearson’s correlation coefficient among model variables for Arusha.**

|  |  | **AM use** | **Vet use** | **No withdraw** |
| --- | --- | --- | --- | --- |
| Antibiotic use | Antibiotic usage | 1.00 |  |  |
|  | Veterinarian (1=use, 0=not use) | -0.24** | 1.00 |  |
| Sectors of vet care | Observe withdrawal from meat and milk | -0.26** | 0.08 | 1.00 |
|  | Vet drug shop | 0.16 | -0.55** | -0.08 |
|  | Traditional healer | -0.01 | -0.13 | -0.09 |
| Herd composition | Small stock daily grazing | 0.57** | -0.31** | -0.15 |
|  | Cattle daily grazing | 0.33** | -0.21** | -0.05 |
|  | Small stock zero grazing | -0.06 | 0.21** | 0.05 |
|  | Cattle zero grazing | -0.20* | 0.54** | -0.13 |
|  | Small stock away grazing | 0.41** | -0.13 | -0.22** |
|  | Cattle away grazing | 0.17* | -0.11 | 0.00 |
| Livestock production | Small stock sold last year | 0.39** | -0.09 | -0.04 |
|  | Cattle sold last year | 0.10 | 0.11 | -0.12 |
|  | Cow milk produced lt/day | -0.14 | 0.14 | 0.00 |
|  | Cow milk sold lt/day | -0.05 | 0.34** | -0.13 |
|  | Frequency beef consumption (0=never, 6=daily) | 0.07 | 0.03 | 0.15 |
|  | Frequency small-stock consumption | 0.02 | -0.07 | 0.05 |
| Agriculture | Acres planted in crops | 0.33** | -0.10 | -0.12 |
|  | Kg of crops sold | -0.01 | 0.03 | -0.19* |
| Modernization | Distance to urban center (km) | 0.38** | -0.60** | 0.09 |
|  | Age (years) | -0.04 | 0.02 | -0.13 |
|  | Education (0=illiterate, 7=post college) | 0.25** | -0.07 | -0.05 |
|  | Owns bike, motorbike or car/truck (1=yes, 0=no) | 0.25** | -0.05 | -0.04 |
|  | House has electricity | -0.03 | 0.05 | 0.06 |
|  | Owns cell phone | 0.14 | -0.04 | -0.05 |
|  | Owns radio | -0.07 | 0.16 | -0.06 |
|  | Has saving account | 0.07 | -0.11 | -0.15 |
| Season | Dry season | -0.07 | -0.10 | -0.26* |

**Table I. Full models combining Maasai and Arusha**. Lay antimicrobial use (AM), veterinary consolation (VC) and observing withdrawal periods (OW).

|  | AM | VC | OW |
| --- | --- | --- | --- |
| Maasai | 1.041*** | 1.193* | 1.675*** |
| Vet drug shop | 0.032 | -3.485*** | 0.475 |
| Traditional Healer | 0.074 | -3.247*** | 1.501 |
| Small stock daily grazing | 0.001** | 0.001 | 0.008** |
| Cattle daily grazing | 0.000 | 0.001 | 0.009 |
| Small stock zero grazing | 0.003 | -0.020 | -0.152 |
| Cattle zero grazing | -0.232*** | 1.256*** | 0.144 |
| Small stock away grazing | -0.000 | 0.002 | 0.029** |
| Cattle away grazing | -0.000 | -0.003 | -0.006 |
| Small stock sold last year | -0.002 | -0.127*** | -0.016 |
| Cattle sold last year | 0.004 | 0.162** | -0.159* |
| Cow milk produced lt/day | -0.000 | 0.029* | -0.030 |
| Cow milk sold lt/day | 0.001 | 0.024 | -0.028 |
| Frequency beef consumption (0=never, 6=daily) | 0.013 | -0.238 | -0.355* |
| Frequency small-stock consumption | 0.030 | 0.338* | 0.194 |
| Acres planted in crops | 0.001 | -0.003 | 0.014 |
| Kg of crops sold | -0.002 | 0.006 | -0.015 |
| Dry season | -0.170* | -0.047 | 0.807* |
| Distance to urban center (km) | 0.000 | 0.003 | 0.009 |
| Age (years) | -0.002 | 0.003 | 0.005 |
| Education (0=illiterate, 7=post college) | 0.043 | -0.105 | -0.168 |
| Owns bike, motorbike or car/truck (1=yes, 0=no) | 0.123 | -0.645 | -0.400 |
| House has electricity | 0.024 | -0.244 | -0.565 |
| Owns cellphone | 0.092 | 0.546 | 1.262* |
| Owns radio | 0.031 | 0.502 | -0.417 |
| Has savings account | -0.019 | -0.827* | 0.032 |
| Constant | 0.032 | -0.591 | -1.654 |
| Pseudo R2 | 0.18 | 0.44 | 0.52 |
| Prob>chi2 | 0.00 | 0.00 | 0.00 |
| Log Likelihood | -499.32 | -108.64 | -85.12 |
| LR chi2 | 216.45 | 171.49 | 183.06 |
| Obs | 291 | 291 | 291 |

Note: *** indicates *P*<0.01, ***P*<0.05, **P*<0.10.

**Table J. Full models for Maasai**. Lay antimicrobial use (AM), veterinary consolation (VC) and observing withdrawal periods (OW).

|  | AM | VC | OW |
| --- | --- | --- | --- |
|  | β | β | β |
| Vet drug shop | -0.024 | -3.903*** | 0.497 |
| Traditional Healer | 0.067 | -3.137*** | 1.386 |
| Small stock daily grazing | 0.001** | 0.001 | 0.006 |
| Cattle daily grazing | 0.000 | 0.002 | 0.014* |
| Small stock zero grazing | 0.062 | -0.388 | -0.133 |
| Cattle zero grazing | -0.191 | 2.390 | -1.013 |
| Small stock away grazing | -0.000 | 0.003 | 0.026 |
| Cattle away grazing | -0.000 | -0.003 | -0.001 |
| Small stock sold last year | -0.003 | -0.110*** | 0.025 |
| Cattle sold last year | 0.006 | 0.117* | -0.250** |
| Cow milk produced lt/day | -0.001 | 0.016 | -0.068* |
| Cow milk sold lt/day | 0.001 | -0.017 | -0.061 |
| Frequency beef consumption (0=never, 6=daily) | 0.001 | -0.183 | -0.112 |
| Frequency small-stock consumption | 0.060 | 0.739*** | 0.122 |
| Acres planted in crops | 0.000 | -0.011 | 0.007 |
| Kg of crops sold | -0.000 | 0.011 | -0.014 |
| Dry season | -0.185* | 0.128 | 0.742 |
| Distance to urban center (km) | 0.001 | 0.001 | 0.002 |
| Age (years) | -0.003 | 0.008 | -0.001 |
| Education (0=illiterate, 7=post college) | 0.001 | -0.157 | -0.156 |
| Owns bike, motorbike or car/truck (1=yes, 0=no) | 0.042 | -0.288 | -0.177 |
| House has electricity | 0.035 | -1.271 | -1.696 |
| Owns cellphone | -0.042 | 2.143** | 1.635 |
| Owns radio | 0.062 | 0.309 | -1.910* |
| Has savings account | 0.010 | -0.646 | -0.039 |
| Constant | 1.286*** | -1.643 | 1.930 |
| Pseudo R2 | 0.04 | 0.43 | 0.44 |
| Prob>chi2 | 0.14 | 0.00 | 0.00 |
| Log Likelihood | -353.58 | -74.55 | -31.24 |
| LR chi2 | 32.78 | 111.57 | 48.69 |
| Obs | 200 | 200 | 200 |

Note: *** indicates *P*<0.01, ***P*<0.05, **P*<0.10.

**Table K. Full models in Arusha**. Lay antimicrobial use (AM), veterinary consolation (VC) and observing withdrawal periods (OW)

|  | AM | VC | OW |
| --- | --- | --- | --- |
|  | β | β | β |
| Vet drug shop | -0.108 | -232.792 | 0.937 |
| Traditional Healer | -15.824* | 0.000 | -12.214 |
| Small stock daily grazing | 0.042*** | -8.464 | -0.168*** |
| Cattle daily grazing | 0.096* | 33.772 | -0.338 |
| Small stock zero grazing | 0.001 | 6.113 | 0.333 |
| Cattle zero grazing | -0.234 | 53.931 | -0.505 |
| Small stock away grazing | 0.014 | -15.771 | -0.225** |
| Cattle away grazing | 0.067* | 23.765 | 0.059 |
| Small stock sold last year | -0.045 | 22.092 | 0.801** |
| Cattle sold last year | -0.078 | -6.361 | -0.345 |
| Cow milk produced lt/day | 0.010 | 0.094 | -0.040 |
| Cow milk sold lt/day | 0.258* | 12.259 | -0.306 |
| Frequency beef consumption (0=never, 6=daily) | 0.247 | -17.925 | 1.039** |
| Frequency small-stock consumption | -0.051 | 4.221 | -0.805 |
| Acres planted in crops | -0.071 | -3.036 | 0.354 |
| Kg of crops sold | -0.007 | -2.305 | -0.123 |
| Dry season | 0.276 | 7.980 | -3.404** |
| Distance to urban center (km) | 0.111 | -31.145 | 0.059 |
| Age (years) | 0.019* | -1.115 | -0.064 |
| Education (0=illiterate, 7=post college) | 0.275** | 38.718 | -0.647* |
| Owns bike, motorbike or car/truck (1=yes, 0=no) | 0.786** | -20.674 | 0.828 |
| House has electricity | 0.483 | 83.749 | 0.904 |
| Owns cellphone | 1.141** | -85.630 | -5.815** |
| Owns radio | -0.383 | 68.446 | -0.001 |
| Has savings account | 0.076 | -24.055 | 1.104 |
| Constant | -4.672*** | 276.601 | 9.909** |
| Pseudo R2 | 0.38 | 1.00 | 0.49 |
| Prob>chi2 | 0.00 | . | 0.00 |
| Log Likelihood | -86.78 | 0.00 | -24.67 |
| LR chi2 | 107.54 | 108.90 | 47.32 |
| Obs | 91 | 91 | 91 |

Note: *** indicates *P*<0.01, ***P*<0.05, **P*<0.10.
