# Supplementary material for: Antimicrobial Use and Veterinary Care among Agro-Pastoralists in Northern Tanzania
Source: PLoS One. 2017 Jan 26;12(1):e0170328. doi: 10.1371/journal.pone.0170328 (PMC5268417; doi:10.1371/journal.pone.0170328)
Supplement: S2 Appendix — (PDF) [file pone.0170328.s002.pdf]

1. Village *

- Mt. Meru
- Nodonjukin
- Loroi
- Meliot

2. Enumerator *

[redacted]

Participant ID Number *

3. Survey Year *

- 2016
- 2015
- 2014
- 2013

Survey Month *

January
February
March
April
May
June
July
August
September
October
November
December

Survey Day *

1
2
3
4
5
6
7
8
9
10
11
12
13
14
15
16
17
18
19
20
21
22
23
24
25
26
27
28
29
30
31

Basic Demographics

4. Gender *

- Male
- Female

5. What is your relationship to the head of the household? *

- Head of household
- Spouse of head of household
- Son of head of household
- In-law
- Hired labor
- Daughter of head of household
- Other

6. Ethnic Group *

- Chagga
- Maasai
- Warusha
- Other

7. How old are you? *

Enter a number.

8. What is your religion? *

- Protestant Christian
- Catholic Christian
- Muslim
- Traditional
- None
- Other

9. Do you read? *

- Yes
- No

10. Highest level of education *

- No formal education
- Some primary school
- Completed primary school
- Some high school
- Completed high school
- Some education beyond high school
- Finished college
- Postgraduate or above

Life History/Demographics

11. Are you married? *

- Yes
- Never
- Divorced
- Widow(er)

12. How many times have you been married? *

- Never
- 1
- 2
- 3
- 4
- 5
- 6
- 7
- 8
- 9
- 10+

13. If married, how old were you when you got married the first time? *
Enter a number.

- 10
- 11
- 12
- 13
- 14
- 15
- 16
- 17
- 18
- 19
- 20
- 21
- 22
- 23
- 24
- 25
- 26
- 27
- 28
- 29
- 30
- 31
- 32
- 33
- 34
- 35
- 36
- 37
- 38
- 39
- 40
- 41
- 42
- 43
- 44
- 45
- 46
- 47
- 48
- 49
- 50
- NEVER MARRIED

14. How many wives do you or your husband have currently? *

- 0
- 1
- 2
- 3
- 4
- 5
- 6
- 7
- 8
- 9
- 10+

15. What is your birth order? *

- First
- Middle
- Last

16. How many siblings do you have? *

	0	1	2	3	4	5	6	7	8	9	10+
Brothers living											
Brothers deceased											
Sisters living											
Sisters deceased											

Children

17. How old were you when your first child was born? *

- No Children
- 10
- 11
- 12
- 13
- 14
- 15
- 16
- 17
- 18
- 19
- 20
- 21
- 22
- 23
- 24
- 25
- 26
- 27
- 28
- 29
- 30
- 31
- 32
- 33
- 34
- 35
- 36
- 37
- 38
- 39
- 40
- 41
- 42
- 43
- 44
- 45
- 46
- 47
- 48
- 49
- 50

18. How many children do you have? *

	0	1	2	3	4	5	6	7	8	9	10	11	12	13	14	15	16+
Sons living *	○	○	○	○	○	○	○	○	○	○	○	○	○	○	○	○	○
Sons deceased *	○	○	○	○	○	○	○	○	○	○	○	○	○	○	○	○	○
Daughters living *	○	○	○	○	○	○	○	○	○	○	○	○	○	○	○	○	○
Daughters deceased *	○	○	○	○	○	○	○	○	○	○	○	○	○	○	○	○	○

19. Did you have any children that didn't survive to one year old? *

○ Yes

○ No

20. Number of persons in household
Enter numbers for all that apply.
If none apply, enter 0 in at least one box.

	Number	Number in School
Children less than school age	Number	Number in School
Females : Primary school age	Number	Number in School
Females : Secondary school age	Number	Number in School
Females : Between 19-65	Number	Number in School
Females : Over 65	Number	Number in School
Males : Primary school age	Number	Number in School
Males : Secondary school age	Number	Number in School
Males : Between 19-65	Number	Number in School
Males : Over 65	Number	Number in School

Health/Health Services

21. What health services do you use for yourself and family? **CHECK ALL THAT APPLY** *

- ☐ Government clinic
- ☐ Private doctor
- ☐ Government hospital
- ☐ Private hospital
- ☐ Local/indigenous practitioners
- ☐ Dispensary
- ☐ None
- ☐ Other (please specify)

22. If you can't treat yourself, where do you go FIRST for medical treatment? *

- Government clinic
- Private doctor
- Government hospital
- Private hospital
- Local/indigenous practitioners
- Dispensary
- None
- Other (please specify)

23. If you go to [repeat what they answered above], and they can't help you, then where do you go next for medical treatment? *

This CANNOT be the SAME SELECTION AS 22!

- Government clinic
- Private doctor
- Govt hospital
- Private hospital
- Local/indigenous practitioners
- None, I only have ONE option
- Other (please specify)

24. What was the most recent illness for a person in the household? *

25. Where did you USUALLY get medicine come from? *

- Government clinic/ hospital
- Private doctor
- Private clinic/ hospital
- Pharmacy
- Friend or neighbor
- N/A
- Other (please specify)

26. What was the most recent exotic medicine someone in your household used? *

- I have not used an exotic medicine recently
- Yes, but I don't know the name
- Yes, I have used the name is?

27. How many times has someone used antibiotics in your household in the last year?

- 0
- 1-3 times
- 4-6 times
- 7-10 times
- Over 10 times

28. Where did you USUALLY get ANTIBIOTICS come from? *

- Government clinic/ hospital
- Private doctor
- Private clinic/ hospital
- Pharmacy
- Friend or neighbor
- N/A
- Other (please specify) ______

29. Are children less than 5 years old in the household currently vaccinated against one or more diseases? *

- Yes
- No
- No children under 5 years

30. How much time does it take to travel to the health center or hospital that you use (ONE WAY)?

Enter numbers. Only need to fill in one box.

minutes by foot ______

minutes by vehicle ______

31. How many visits to a health clinic or hospital were made by household members in the last 3 months (sum of everyone in house)? *

- 0-1 time
- 2-4 times
- 5-10 times
- 11-20 times
- 20+ times

Social - Household Information

32. How many dwellings? *

Enter numbers.

- 1-3
- 3-6
- 7-10
- Over 10

33. Animal House

	Yes	No
Cattle		
Goats and Sheep		
Chicken		
Dog		

34. The house of the respondent is: *

- On own land
- On communal land
- Other

35. Toilet location? *

- Inside the house
- Outside the house
- Both
- None

36. What type of toilet? *

- Flush toilet
- Pit toilet
- Trench
- Bush in dedicated location
- Bush in general
- Other

37. Electricity –Household inventory *

- None
- Grid (this means a power line is connected to house
- Off grid (but available via local production such as solar panels)

38. How many of these does your household have? *

	0	1	2	3	4	5+
Radio Functioning						
Television Functioning						
Refrigerator Functioning						
Bicycle : Functioning						
Motorcycle/Scooter : Functioning						
Car/Truck : Functioning						
Cell Phone : Functioning						
Horse Cart: Functioning						
Plough : Functioning						
Syringe : Functioning						
Needles : Functioning						
M'kokoteli(Wheel Barrow)						

39. Where do you get your water? **CHECK ALL THAT APPLY** *

- Communal well
- Private well
- River water
- Lake or water hole
- Rain water or seasonal stream
- Household Stand pipe
- Nieghbor Standpipe
- Pay for delivery
- Cistern
- Other (please specify)

40. Where do you usually get your water? *

- Communal well
- Private well
- River water
- Lake or impoundment
- Rain water seasonal stream
- Household Stand pipe
- Neighbor Standpipe
- Pay for delivery
- Other (please specify)

41. Do livestock or wildlife drink from the same water source? For example, livestock and humans all drink from standpipe *

- No, water only for people
- Livestock drink the same water
- Wildlife drink the same water
- Both livestock and wildlife drink the same water

42. Do you treat drinking water? *

- No treatment
- Boil every time
- Boil some of the time
- Chemical treatment
- Filter
- Other

Consumption

43. How often do you eat meat in your meals? *

	Every meal	Once per day	More than once a week	Less than once per week	Less than once per month	Special occasions	Never
Beef *							
Sheep *							
Goat *							
Pig meat *							
Poultry *							

44. Quantity of cow's milk consumed by household per day

	Quantity
Home produced milk	Quantity
Purchased milk	Quantity
Home produced butter	Quantity
Purchased butter	Quantity
Home produced eggs	Quantity
Purchased eggs	Quantity

Economic - Crop Enterprise, Land

45. **FOR THE LAST HARVEST** what types of crops did you grow?

If NO CROPS, enter NA for at least one entry.

	Crop name	Area planted	Draught power used (Yes/No)	Draught power used (Type)	Fertilizer used (Yes/No)	Fertilizer used (Type)	Crop output use (Home Consumption)
Crop 1	Crop name	Area planted	Draught power used (Yes/No)	Draught power used (Type)	Fertilizer used (Yes/No)	Fertilizer used (Type)	Crop output use (Home Consumption)
Crop 2	Crop name	Area planted	Draught power used (Yes/No)	Draught power used (Type)	Fertilizer used (Yes/No)	Fertilizer used (Type)	Crop output use (Home Consumption)
Crop 3	Crop name	Area planted	Draught power used (Yes/No)	Draught power used (Type)	Fertilizer used (Yes/No)	Fertilizer used (Type)	Crop output use (Home Consumption)
Crop 4	Crop name	Area planted	Draught power used (Yes/No)	Draught power used (Type)	Fertilizer used (Yes/No)	Fertilizer used (Type)	Crop output use (Home Consumption)
Crop 5	Crop name	Area planted	Draught power used (Yes/No)	Draught power used (Type)	Fertilizer used (Yes/No)	Fertilizer used (Type)	Crop output use (Home Consumption)

46. Total crop production expenditures for the most recent complete growing season
If NO CROPS, enter 0 for at least one entry.

	Cost (in TZS)
Seed	Cost (in TZS)
Fertilizer	Cost (in TZS)
Herbicide	Cost (in TZS)
Pesticide	Cost (in TZS)
Tractor Rental	Cost (in TZS)
Other 1 (specify below)	Cost (in TZS)
Other 2 (specify below)	Cost (in TZS)
Other 3 (specify below)	Cost (in TZS)

47. Did you lose 1/4 or more of your crops in the following years? *

2014	Yes, I lost 1/4 of my crops<br>Yes, I lost 1/2 of my crops<br>Yes, I lost 3/4 of my crops<br>Yes, I lost ALL of my crops<br>No, I did not lose any crops<br>No, I don't GROW ANY CROPS<br>I don't remember
2013	Yes, I lost 1/4 of my crops<br>Yes, I lost 1/2 of my crops<br>Yes, I lost 3/4 of my crops<br>Yes, I lost ALL of my crops<br>No, I did not lose any crops<br>No, I don't GROW ANY CROPS<br>I don't remember
2012	Yes, I lost 1/4 of my crops<br>Yes, I lost 1/2 of my crops<br>Yes, I lost 3/4 of my crops<br>Yes, I lost ALL of my crops<br>No, I did not lose any crops<br>No, I don't GROW ANY CROPS<br>I don't remember
2011	Yes, I lost 1/4 of my crops<br>Yes, I lost 1/2 of my crops<br>Yes, I lost 3/4 of my crops<br>Yes, I lost ALL of my crops<br>No, I did not lose any crops<br>No, I don't GROW ANY CROPS<br>I don't remember
2010	Yes, I lost 1/4 of my crops<br>Yes, I lost 1/2 of my crops<br>Yes, I lost 3/4 of my crops<br>Yes, I lost ALL of my crops<br>No, I did not lose any crops<br>No, I don't GROW ANY CROPS<br>I don't remember

48. If you lost a 1/4 or more of your crops in the following years, what was the major reason? *

2014

- Drought
- Pests or Disease
- Bad Seeds or Not Enough Fertilizer
- Animals Ate
- Didn't take care of the field
- I don't have crops
- I didn't lose any crops that year
- I don't remember

2013

- Drought
- Pests or Disease
- Bad Seeds or Not Enough Fertilizer
- Animals Ate
- Didn't take care of the field
- I don't have crops
- I didn't lose any crops that year
- I don't remember

2012

- Drought
- Pests or Disease
- Bad Seeds or Not Enough Fertilizer
- Animals Ate
- Didn't take care of the field
- I don't have crops
- I didn't lose any crops that year
- I don't remember

2011

- Drought
- Pests or Disease
- Bad Seeds or Not Enough Fertilizer
- Animals Ate
- Didn't take care of the field
- I don't have crops
- I didn't lose any crops that year
- I don't remember

2010

- Drought
- Pests or Disease
- Bad Seeds or Not Enough Fertilizer
- Animals Ate
- Didn't take care of the field

I don't have crops
I didn't lose any crops that year
I don't remember

Livestock Enterprise, Assets

49. Cattle
Enter numbers.
If NO CATTLE, enter 0 for at least one entry.

	Adult (Number)	Young (Number)
Kept in a boma in homestead part of all of the day	Adult (Number)	Young (Number)
Moves in and out of the homestead	Adult (Number)	Young (Number)
Herded away from the homestead	Adult (Number)	Young (Number)

50. Sheep & goats

Enter numbers.

If NO GOATS AND SHEEP, enter 0 for at least one entry.

	Adult (Number)	Young (Number)
Kept in a boma in homestead part of all of the day	Adult (Number)	Young (Number)
Moves in and out of the homestead	Adult (Number)	Young (Number)
Herded away from the homestead	Adult (Number)	Young (Number)

51. Donkeys

Enter numbers.

If NO DONKEYS, enter 0 for at least one entry.

	Adult (Number)	Young (Number)
Kept in a boma in homestead part of all of the day	Adult (Number)	Young (Number)
Moves in and out of the homestead	Adult (Number)	Young (Number)
Herded away from the homestead	Adult (Number)	Young (Number)

52. Pigs
Enter numbers.
If NO PIGS, enter 0 for at least one entry.

	Animals (Number)	Purchase in last year? If Yes, put number	Source of Purchase
adult	Animals (Number)	Purchase in last year? If Yes, put number	Source of Purchase
young	Animals (Number)	Purchase in last year? If Yes, put number	Source of Purchase

53. Poultry system

	Birds (Number)	Housing?	Purchase in last year, if yes put #
Free range	Birds (Number)	Housing?	Purchase in last year, if yes put #

54. number of eggs produced per flock/day? *
Enter a number.

eggs/flock/day

55. Average amount of eggs sold by the household *
Enter a number.

eggs per day

56. How many animals have you purchased in the last year? *

Cattle ______

Sheep/goats ______

Donkeys ______

57. How many animals have you sold in the last year? *

Cattle ______

Sheep/goats ______

Donkeys ______

58. Where do you sell cattle and small livestock? **(Check ALL that apply)** *

- [ ] Local market
- [ ] Regional market
- [ ] Neighbors
- [ ] Butcher/abattoir
- [ ] I don't sell cattle
- [ ] I don't own any cattle
- [ ] Other (specify) ______

59. When you sell livestock how far do you usually travel? *

- 1 hour or less
- More than 1 hour less than 1 day
- More than 1 day
- More than 2 days
- N/A

60. When you purchase livestock how far do you usually travel? *

- 1 hour or less
- More than 1 hour less than 1 day
- More than 1 day
- More than 2 days
- N/A

61. Does someone outside your household manage your livestock for you? *

- Yes
- No

62. If someone else manages your livestock outside of your household, why? *

- Too many to manage alone
- Other person needed livestock for milk
- N/A
- Other (specify) ______

63. How many animals do you currently manage for someone else? *
Enter numbers for all that apply.
If none apply, enter 0 for at least one entry.

Cattle ______
Goats ______
Sheep ______
Donkeys ______
Horses ______
Other ______

64. Why are you keeping stock for someone else? (SKIP IF NOT KEEPING STOCK FOR SOMEONE ELSE) *

- Owner had too many to manage alone
- Needed livestock for milk
- Kumharia
- N/A
- Other (specify) ______

65. How much land does the household graze and not share with other livestock holders for grazing? *

Enter the number and the units.

- ○ Yes, write in number and units ________ *
- ○ None

66. Does your family have access to common grazing land? *

- ○ Yes
- ○ No

67. What factors keep you from increasing your herd size? (CHECK ALL THAT APPLY) *

- ☐ Lack of money
- ☐ Lack of land
- ☐ Lack of water
- ☐ Lack of labor
- ☐ Don't want any more
- ☐ Too expensive to maintain
- ☐ Disease limitations
- ☐ N/A
- ☐ Other (specify) ________

68. How often do your livestock come into contact with livestock from other villages/communities when grazing? *

- Never
- Every day
- Once or more per week
- Once or more per month
- Less than once per month

Cattle Management

69. How many times in a normal year do you move your livestock? *

- 0
- 1
- 2
- 3
- 4
- 5
- 6+
- NA, I have no livestock

70. How many times do you move your livestock in a dry year? *

- 0
- 1
- 2
- 3
- 4
- 5
- 6+
- NA, I have no livestock

71. How far do you move your livestock in a normal year? *

- Less than one day walk
- One to two days
- Three to four days
- More than four days
- N/A

72. How far do you move your livestock in a dry year? *

- Less than one day walk
- One to two days
- Three to four days
- More than four days
- N/A

73. Is this year a dry year, normal year, or wet year? *

- Dry year
- Normal year
- Wet year

Economic - Livestock Health and Loss

74. Did you lose one-quarter or more of your livestock in the following years? *

For example, if their herd was 4 and they lost 1 cow or sheep then say "yes"

Year	Response options
2014	Yes, I lost 1/4 of my herd<br>Yes, I lost 1/2 of my herd<br>Yes, I lost 3/4 of my herd<br>Yes, I lost ALL of my herd<br>No, I didn't lose more than 1/4<br>No, I don't have any livestock<br>I don't remember
2013	Yes, I lost 1/4 of my herd<br>Yes, I lost 1/2 of my herd<br>Yes, I lost 3/4 of my herd<br>Yes, I lost ALL of my herd<br>No, I didn't lose more than 1/4<br>No, I don't have any livestock<br>I don't remember
2012	Yes, I lost 1/4 of my herd<br>Yes, I lost 1/2 of my herd<br>Yes, I lost 3/4 of my herd<br>Yes, I lost ALL of my herd<br>No, I didn't lose more than 1/4<br>No, I don't have any livestock<br>I don't remember
2011	Yes, I lost 1/4 of my herd<br>Yes, I lost 1/2 of my herd<br>Yes, I lost 3/4 of my herd<br>Yes, I lost ALL of my herd<br>No, I didn't lose more than 1/4<br>No, I don't have any livestock<br>I don't remember
2010	Yes, I lost 1/4 of my herd<br>Yes, I lost 1/2 of my herd<br>Yes, I lost 3/4 of my herd<br>Yes, I lost ALL of my herd<br>No, I didn't lose more than 1/4<br>No, I don't have any livestock<br>I don't remember

75. If you lost 1/4 or more of your herds in the following years, what was the major reason? *

2014	Drought<br>Pests or Disease<br>I did not lose 1/4 of my herd that year<br>I don't have any livestock<br>I don't remember
2013	Drought<br>Pests or Disease<br>I did not lose 1/4 of my herd that year<br>I don't have any livestock<br>I don't remember
2012	Drought<br>Pests or Disease<br>I did not lose 1/4 of my herd that year<br>I don't have any livestock<br>I don't remember
2011	Drought<br>Pests or Disease<br>I did not lose 1/4 of my herd that year<br>I don't have any livestock<br>I don't remember
2010	Drought<br>Pests or Disease<br>I did not lose 1/4 of my herd that year<br>I don't have any livestock<br>I don't remember

76. What animal health services do you use for livestock? **CHECK ALL THAT APPLY** *

- ☐ Ag extension officer
- ☐ Government vet
- ☐ Private vet
- ☐ Drug shop
- ☐ Indigenous healer
- ☐ None
- ☐ Other (specify) ____

77. Describe the most recent illness in your herd (SKIP IF NO RECENT ILLNESS)

	Animal	Illness	Symptoms	Treatment	Duration	Number sick	Recovered
Most recent	Animal	Illness	Symptoms	Treatment	Duration	Number sick	Recovered
Next most recent	Animal	Illness	Symptoms	Treatment	Duration	Number sick	Recovered

78. Are your animals vaccinated against any diseases? (SKIP IF NO ANIMALS ARE VACCINATED)

List for which diseases animals are vaccinated.

	Disease 1	Disease 2	Disease 3	Disease 4	Other diseases
Cattle	Disease 1	Disease 2	Disease 3	Disease 4	Other diseases
Smallstock	Disease 1	Disease 2	Disease 3	Disease 4	Other diseases
Dogs	Disease 1	Disease 2	Disease 3	Disease 4	Other diseases

79. Where do you get vaccines for livestock? *

- Veterinarian
- Animal drug shop
- Agricultural extension
- Friends
- N/A, the HOUSEHOLD does not purchase vaccines
- Don't Know
- Other (specify)

80. Number of calves born dead in the last year from sick cows? *

Enter a number.

81. Number of kids born dead in last year from sick mother? *
Enter a number.

82. How do you dispose of a dead animal? *

- Eat
- Skin and bury
- Bury without skinning
- Leave it
- Burn it
- Cook for dogs and pigs
- N/A

83. When you treat an animal do you use the milk or meat from it? *

- Immediately
- After 3 days
- After 10 days
- When the animal is healthy
- N/A

84. Has sickness in your herd reduced the time available for other business/work in the last year? *

- Yes
- No

85. Has any family member become sick or died because of eating product from sick animal? *

- Yes
- No

Exotic Medicines for Livestock

86. Do you keep exotic medicines FOR LIVESTOCK at home? *

- Yes
- No

87. What exotic medicines **FOR LIVESTOCK** do you keep on hand at home?

	Medicine	Brand	Where it was purchased	When it was purchased	Use by date (EXPIRATION)
Medicine 1	Medicine	Brand	Where it was purchased	When it was purchased	Use by date (EXPIRATION)
Medicine 2	Medicine	Brand	Where it was purchased	When it was purchased	Use by date (EXPIRATION)
Medicine 3	Medicine	Brand	Where it was purchased	When it was purchased	Use by date (EXPIRATION)
Medicine 4	Medicine	Brand	Where it was purchased	When it was purchased	Use by date (EXPIRATION)
Medicine 5	Medicine	Brand	Where it was purchased	When it was purchased	Use by date (EXPIRATION)
Medicine 6	Medicine	Brand	Where it was purchased	When it was purchased	Use by date (EXPIRATION)
Medicine 7	Medicine	Brand	Where it was purchased	When it was purchased	Use by date (EXPIRATION)
Medicine 8	Medicine	Brand	Where it was purchased	When it was purchased	Use by date (EXPIRATION)

88. Where do you get exotic medicines for livestock? *

- ○ Veterinarian
- ○ Animal drug shop
- ○ Agricultural extension
- ○ Friends
- ○ N/A, the HOUSEHOLD does not purchase exotic medicines
- ○ Other (specify) ______

Cattle Sickness

89. What do you do to avoid disease/outbreak in livestock? **CHECK ALL THAT APPLY** *

Select all that apply.

- ☐ Keep calves separate
- ☐ Make a shed or pen to prevent contact from other domestic and wild animals
- ☐ Keep new cattle separately
- ☐ Graze sick cattle separately
- ☐ Feed supplementation
- ☐ Buy new cattle from local area
- ☐ Vaccination
- ☐ Treatment (Animal Health)
- ☐ Treatment (traditional)
- ☐ Spraying
- ☐ Do nothing
- ☐ Other (specify) ______

90. Do you change where you graze when your livestock to avoid sickness? *

- Yes
- No
- N/A, I don't have any cows
- N/A, my cows are zero grazing

91. Do you ever give your livestock medicine? *

- Yes, I give them exotic and traditional medicine
- Yes, but I only give them traditional medicine NOT exotic medicine
- Yes, but I only give them exotic medicine NOT traditional medicine
- No, I do not give them any medicine.

92. How much do you use per dose for a full grown cow?

	Length of treatment (days)	dose for adult cow	Don't Know, Vet gives drugs
Pen-strep	Length of treatment (days)	dose for adult cow	Don't Know, Vet gives drugs
Basulfa	Length of treatment (days)	dose for adult cow	Don't Know, Vet gives drugs
Bamisola	Length of treatment (days)	dose for adult cow	Don't Know, Vet gives drugs
Dininabazen	Length of treatment (days)	dose for adult cow	Don't Know, Vet gives drugs
Parvexon	Length of treatment (days)	dose for adult cow	Don't Know, Vet gives drugs
Alfamec	Length of treatment (days)	dose for adult cow	Don't Know, Vet gives drugs
Ivermectine	Length of treatment (days)	dose for adult cow	Don't Know, Vet gives drugs
Inomazene	Length of treatment (days)	dose for adult cow	Don't Know, Vet gives drugs

(untitled)

93. Does anyone in the household milk cows *

- Yes
- No

94. How much milk do you get from **ONE COW** in the

Dry Season

- Under 1 liter
- 1 liter
- 1-2 liter
- 2 liters
- more than 2 liters

Wet Season

- Under 1 liter
- 1 liter
- 1-2 liter
- 2 liters
- more than 2 liters

95. How many cows does THE HOUSEHOLD milk in the

HOUSEHOLD INCLUDES ALL WIVES

Dry Season

- 1-2
- 2-4
- 5-10
- 11-20
- 21-40
- 41-60
- Over 60

Wet Season

- 1-2
- 2-4
- 5-10
- 11-20
- 21-40
- 41-60
- Over 60

96. Average amount of cow milk sold by the household *
Enter a number.

number of liters/day

97. Average amount of butter sold by the household *
Enter a number.

liters/ market day

98. Does the housheold milk goats/sheep *

- Yes
- No

99. How much milk do you get from **ONE GOAT/SHEEP** in the:

Dry Season

- Under 1 liter
- 1 liter
- 1-2 liter
- 2 liters
- more than 2 liters

Wet Season

- Under 1 liter
- 1 liter
- 1-2 liter
- 2 liters
- more than 2 liters

100. How many goats/sheep does THE HOUSEHOLD milk in the

HOUSEHOLD INCLUDES ALL WIVES

Dry Season

- 1-2
- 2-4
- 5-10
- 11-20
- 21-40
- 41-60
- Over 60

Wet Season

- 1-2
- 2-4
- 5-10
- 11-20
- 21-40
- 41-60
- Over 60

101. Average amount of goat milk sold by the household *

Enter a number.

[ ] liters/ day

102. Where do you normally sell milk or butter?

- [ ] Local market
- [ ] Regional market
- [ ] Neighbors
- [ ] Shop keeper
- [ ] N/A
- [ ] Other (specify) ____

(untitled)

103. Select YES if anyone in the household milk cows OR goats/sheep

- Yes
- No

104. How many times a day do you milk cows or goats?

- 1
- 2
- 3
- 4 or more

105. Do you clean the enyewa of the cows/sheep/goats before milking?

- Yes
- No

106. Do you DRINK/COOK with MILK from? *

SICK COWS

- Yes
- No
- N/A, I don't have any milk producing livestock

SICK GOATS/SHEEP

- Yes
- No
- N/A, I don't have any milk producing livestock

107. Do you SELL MILK from? *

SICK COWS
- Yes
- No
- N/A, I don't have any milk producing livestock

SICK GOATS/SHEEP
- Yes
- No
- N/A, I don't have any milk producing livestock

108. Do you stop selling or consuming milk during treatment with exotic medicines *

- Yes
- No
- N/A

109. Is there a decrease in milk production from sick cows compared to healthy cows? *

- Yes
- No
- No milking of sick cows
- N/A , I don't have any cows

110. Referring to the previous question, If yes, by how much? (SKIP IF NO DECREASE IN MILK PRODUCTION) *

Enter a number.

[ ] liters/day

111. Do you have seperate COLLECTION CONTAINERS for DIFFERENT COWS/SHEEP?
GOATS?

- YES, each animal has a different container
- NO, I use the same container for many animals
- No, I onle have ONE milking animal

112. If one of your milking animals is sick, do you stop milking and clean the collection container BEFORE milking any other animals?

- Yes, every time
- Yes, but only some of the times
- No

113. How often do you clean your calabash/container used for COLLECTING cow and sheep milk

- Only in the morning
- Only in the evening
- In the morning and in the evening
- A few times per week
- I never clean my collection containers

114. What do you clean your milk containers with? **CHECK ALL THAT APPLY**

	Water	Cow Urine
Collection Containers	☐	☐
Storage Containers	☐	☐

115. What CONTAINER do you STORE milk in? **CHECK ALL THAT APPLY**

- [ ] Calabash
- [ ] Plastic Container
- [ ] Metal Pots
- [ ] Other (required) ______ *

116. How many hours do you USUALLY store milk after morning or evening milking before it is all gone?

- ○ Under 1 hr
- ○ 1-2
- ○ 3-4
- ○ 5-7
- ○ 8-12
- ○ 12-24
- ○ More than one day

117. How many times do you bring the milk to boil?

- ○ 1
- ○ 2
- ○ 3
- ○ 4
- ○ Over 4

118. Do you boil milk before putting in calabash to make sour milk?

- Yes, always
- Yes, but only some of the itme
- No, I do not boil the milk
- I do not make sour milk

119. How many days do you keep sour milk before cleaning the calabash?

- Under 1 day
- About 1 day
- 1-2 days
- 3-4 days
- 5-6 days
- A week or more
- I do not make sour milk

120. If you do not boil all of the milk, why do you not boil all of the milk? **CHECK ALL THAT APPLY**

- I do not boil the milk I sell
- Boiled milk tastes bad
- Boiled milk hurts the vitamines in milk
- I do not boil milk
- Other (required) ____ *

121. Do you use the SAME CONTAINERS to collect blood milk and urine

- Yes
- No, I use different containers
- No, I do not collect blood or urine from animals

DAAAGS

122. Do you own any dogs?

- Yes, put how many ______ *
- No

123. How often do you feed your dogs?

- almost everyday
- a few times per week
- a few times per week
- I never feed them, they find food for themselves

124. If you feed your dogs, what do you feed them. Ask SEPERATELY FOR DRY AND WET SEASONS. CHECK ALL THAT APPLY

	Porridge with Milk	Porridge with Water	Milk Water Mixed	Only Milk	Only Water	Left over human food
Dry Season	☐	☐	☐	☐	☐	☐
Wet Season	☐	☐	☐	☐	☐	☐

125. How long do you cook the porridge for dogs?

- ○ Under 5 minutes
- ○ 5-10 minutes
- ○ over 10 minutes

126. Do you cook your porridge for dogs FOR LESS time than you cook porridge for humans?

- ○ Yes
- ○ No

127. If you cook porridge for dogs FOR LESS time, why?

[ ]

128. If you don't drink milk from sick cows, do you still give it to dogs?

- ○ Yes
- ○ No

129. Have you ever seen dogs EAT the poop of. CHECK ALL THAT APPLY

- ☐ Humans
- ☐ Other Dogs
- ☐ Cattle
- ☐ Sheep/Goat

Ego Network

130. In the last year, how many DIFFERENT people did you RECEIVE livestock from. This includes buying, loans, marriage payments, and gifts *

131. In the last year, about how many DIFFERENT people did you GIVE livestock to. This includes selling, loans, marriage payments, and gifts *

132. Please think about the last three times you BOUGHT OR SOLD cattle, who did you BUY or SELL them too *

Exchange 1
- Kin
- Friend
- Livestock Trader
- NA

Exchange 2
- Kin
- Friend
- Livestock Trader
- NA

Exchange 3
- Kin
- Friend
- Livestock Trader
- NA

133. List up to 3 water sources your livestock use in a year.

	Name of Source
Water A	Name of Source
Water B	Name of Source
Water C	Name of Source

134. How often do your livestock use the sources

Water A
- almost everyday
- almost once per week
- about once per month

Water B
- almost everyday
- almost once per week
- about once per month

Water C
- almost everyday
- almost once per week
- about once per month

135. Do your livestock come in contact with other livestock at these sources

Water A
- Yes, many other animals
- Yes, but only a few households
- No, this source is in the household
- No, I take water from source and bring to cattle

Water B
- Yes, many other animals
- Yes, but only a few households
- No, this source is in the household
- No, I take water from source and bring to cattle

Water C
- Yes, many other animals
- Yes, but only a few households
- No, this source is in the household
- No, I take water from source and bring to cattle

136. List up to 3 markets you have bought livestock at

	Name of Source
Market A	Name of Source
Market B	Name of Source
Market C	Name of Source

137. For the year, how many cattle did you BUY at the market

Market A	0<br>1<br>2-4<br>5-10<br>10-30<br>31-50<br>Over 50
Market B	0<br>1<br>2-4<br>5-10<br>10-30<br>31-50<br>Over 50
Market C	0<br>1<br>2-4<br>5-10<br>10-30<br>31-50<br>Over 50

138. For the year, how many cattle did you SELL at the market

Market A
- 0
- 1
- 2-4
- 5-10
- 10-30
- 31-50
- Over 50

Market B
- 0
- 1
- 2-4
- 5-10
- 10-30
- 31-50
- Over 50

Market C
- 0
- 1
- 2-4
- 5-10
- 10-30
- 31-50
- Over 50

Information on wage labor

139. In the last three years, has anyone in the household been employed in wage labor? This means the person is paid at an hourly rate *

- Yes
- NO

140. Please think of the last three people IN THE HOUSEHOLD who worked wage labor. Who?

Person 1

- No person has worked wage labor
- Household Head
- Spouse of Household Head
- Child of household head
- Sibling of Household Head
- Parents of Household Head
- Cousin
- Friend

Person 2

- No person has worked wage labor
- Household Head
- Spouse of Household Head
- Child of household head
- Sibling of Household Head
- Parents of Household Head
- Cousin
- Friend

Person 3

- No person has worked wage labor
- Household Head
- Spouse of Household Head
- Child of household head
- Sibling of Household Head
- Parents of Household Head
- Cousin
- Friend

141. Where did the people go for wage labor?

Person 1
- close to the village
- In an urban area
- many kilometers away

Person 2
- close to the village
- In an urban area
- many kilometers away

Person 3
- close to the village
- In an urban area
- many kilometers away

142. How long did the people work in wage labor?

Person 1
- A few weeks
- A few months
- Many months

Person 2
- A few weeks
- A few months
- Many months

Person 3
- A few weeks
- A few months
- Many months

143. What type of wage labor did the person do. For example, security, work for tourist company, get paid to weed field

Person 1 ________

Person 2 ________

Person 3 ________

144. In the last three years, has anyone in the household made money from self-employment, such as boda-boda, selling charcoal, or selling matunda from garden? *

- ○ Yes
- ○ NO

145. Please think of the last three people IN THE HOUSEHOLD who worked self-employed. Who were they?

Person 1
- Household Head
- Spouse of Household Head
- Child of household head
- Sibling of Household Head
- Parents of Household Head
- Cousin
- Friend

Person 2
- Household Head
- Spouse of Household Head
- Child of household head
- Sibling of Household Head
- Parents of Household Head
- Cousin
- Friend

Person 3
- Household Head
- Spouse of Household Head
- Child of household head
- Sibling of Household Head
- Parents of Household Head
- Cousin
- Friend

146. What type of self-employment did the person do? For example, drive a boda-boda, sell charcoal, or or vegetables/eggs from garden

Person 1 ________

Person 2 ________

Person 3 ________

147. In the last three years, has anyone IN THE HOUSEHOLD made money from salary labor? Salay Labor means paid per month, for example, working for the government *

- Yes
- NO

148. Please think of the last three people who worked salary labor IN THE HOUSEHOLD, who were they?

Person 1

- No person has worked in salary labor
- Household Head
- Spouse of Household Head
- Child of household head
- Sibling of Household Head
- Parents of Household Head
- Cousin
- Friend

Person 2

- No person has worked in salary labor
- Household Head
- Spouse of Household Head
- Child of household head
- Sibling of Household Head
- Parents of Household Head
- Cousin
- Friend

Person 3

- No person has worked in salary labor
- Household Head
- Spouse of Household Head
- Child of household head
- Sibling of Household Head
- Parents of Household Head
- Cousin
- Friend

149. How often do the people work in salary labor?

Person 1
A few weeks
A few months
Many months

Person 2
A few weeks
A few months
Many months

Person 3
A few weeks
A few months
Many months

150. What type of salary labor did the person do. For example, work for government or NGO

Person 1

Person 2

Person 3

151. Has any person working in SALARY or WAGE LABOR AWAY FROM THE VILLAGE, sent money back to the household? *

Person 1

- No person has sent back money
- Household Head
- Spouse of Household Head
- Child of household head
- Sibling of Household Head
- Parents of Household Head
- Cousin
- Friend

Person 2

- No person has sent back money
- Household Head
- Spouse of Household Head
- Child of household head
- Sibling of Household Head
- Parents of Household Head
- Cousin
- Friend

Person 3

- No person has sent back money
- Household Head
- Spouse of Household Head
- Child of household head
- Sibling of Household Head
- Parents of Household Head
- Cousin
- Friend

152. If someone working in wage or salary labor away from household sent money home, how often did they send home?

Person 1	almost every week<br>almost every month<br>only a few times a year
Person 2	almost every week<br>almost every month<br>only a few times a year
Person 3	almost every week<br>almost every month<br>only a few times a year

153. What is the total household wage income per month on average from labor, INCLUDING WAGE, SALARY. SMALL BUSINESS and NOT from livestock or crops? *

Enter a number.

- 0
- 1- 25,000
- 25,000-100,000
- 100,000-200,000
- 200,000-400,000
- 400,000-700,000
- 700,000-1,000,000
- Over 1 million
- Does not want to answer
- Does not know

154. Does any household member maintain a savings account? *

- Bank
- Mpesa
- None

155. What is the current household savings balance? (SKIP IF NO BANKING ACCOUNT) *
Enter a number.

- 0
- 1- 25,000
- 25,000-100,000
- 100,000-200,000
- 200,000-400,000
- 400,000-700,000
- 700,000-1,000,000
- Over 1 million
- Does not want to answer
- Does not know

156. In the LAST YEAR did you receive any support for feeding the household from any NGO/Government? *

- Yes, write in name of source (agency, NGO) ____________
- No

157. Has any household member taken **a loan or loans** in the last 2 years? *

- Yes
- No
- Don't know

158. Why were the loan (s) taken? **CHECK ALL THAT APPLY** *

- Buy food
- Housing
- Transportation
- School fees
- Health care
- Livestock purchase
- Business
- N/A
- Other

Diversification

159. In the last 3 years, have you changed **the size** of your herd or **species composition** because of changes in the environment, such as climate changes. *

- Yes
- No
- No, I did not own any livestock in the past three years

160. In the last 3 years, have you increased the amount of livestock you sold? *

- Yes
- No
- No, I did not own any livestock in last three years

161. In the last 3 years, have you changed the types of crops you grow because of climate changes or to make more money? *

- Yes, because of climate change
- Yes, to make more money
- Yes, because of climate change AND money
- No
- No, I did not have any crops in last 3 years

162. In the last 3 years, have you increased inputs (fertilizer, labor) to increase agricultural output (yield)? *

- Yes
- No
- No, I did not have any crops in last 3 years

Diversification

163. In the Last Year What percent of household income was from Livestock and Crops OR Labor/Assistance *

- 100% Livestock and Crops
- 90% Livestock and Crops and 10% Labor
- 75% Livestock and Crops and 25% Labor
- 50% Livestock and Crops and 50% Labor
- 25% Livestock and Crops and 75% Labor
- 10% Livestock and Crops and 90% Labor
- 100% Labor and assistance

164. From the money earned by livestock and crops, what percent is from livestock and from crops *

- 100% Livestock
- 90% Livestock and 10% Crops
- 75% Livestock and 25% Crops
- 50% Livestock and 50% Crops
- 25% Livestock and 75% Crops
- 10% Livestock and 90% Crops
- 100% Crops
- NA, I get ALL income from Labor/Assistance

VERIFY

165. Did you get a GPS point and label the point the household id number *

- Yes
- NO

166. Did you get a milk sample (fresh, sour, cattle and goat) if they have this milk? If they did have milk, make sure you put ID Number, Date, Time the Milking (morning or evening), Time you collected, and Type of Container used (calabash, plastic container) *

- Yes
- No

167. Did you get poop samples (human, cattle, sheep, dog, chicken, donkey) and make sure to put ID, DATE, Time of COllection, Species and OPEN/ENCLOSURE? *

- Yes
- No
